# The power and limits of predicting inter-protein exon-exon interactions using protein 3D structures

**DOI:** 10.1093/bioadv/vbag032

**Published:** 2026-01-27

**Authors:** Jeanine Liebold, Aylin Del Moral-Morales, Karen Manalastas-Cantos, Olga Tsoy, Stefan Kurtz, Jan Baumbach, Khalique Newaz

**Affiliations:** Institute for Computational Systems Biomedicine, Universität Hamburg, Hamburg 22761, Germany; Center for Bioinformatics, Faculty of Mathematics, Informatics and Natural Sciences, Universität Hamburg, Hamburg 22761, Germany; Institute for Computational Systems Biomedicine, Universität Hamburg, Hamburg 22761, Germany; Departamento de Ciencias Naturales, Universidad Autónoma Metropolitana-Cuajimalpa, Mexico City 05348, Mexico; Institute for Computational Systems Biomedicine, Universität Hamburg, Hamburg 22761, Germany; Leibniz-Institut für Virologie (LIV), Centre for Structural Systems Biology (CSSB), Hamburg 22607, Germany; Center for Data and Computing in Natural Sciences, Universität Hamburg, Hamburg 22761, Germany; Institute for Computational Systems Biomedicine, Universität Hamburg, Hamburg 22761, Germany; Department of Computer Science, Bioinformatics, Vrije Universiteit Amsterdam, Amsterdam 1081, The Netherlands; Center for Bioinformatics, Faculty of Mathematics, Informatics and Natural Sciences, Universität Hamburg, Hamburg 22761, Germany; Institute for Computational Systems Biomedicine, Universität Hamburg, Hamburg 22761, Germany; Department of Mathematics and Computer Science, University of Southern Denmark, Odense 5230, Denmark; Institute for Computational Systems Biomedicine, Universität Hamburg, Hamburg 22761, Germany; Center for Data and Computing in Natural Sciences, Universität Hamburg, Hamburg 22761, Germany

## Abstract

**Motivation:**

Alternative splicing (AS) effects on cellular functions can be captured by studying changes in the underlying protein-protein interactions (PPIs). Because AS results in the gain or loss of exons, existing methods for predicting AS-related PPI changes utilize known inter-protein exon-exon interactions (EEIs), which cover <0.5% of known human PPIs. Hence, there is a need to extend the limited EEI knowledge to advance the functional understanding of AS. Here, we explore whether existing 3-dimensional (3D) protein structure-based computational PPI interface prediction (PPIIP) methods, originally designed to predict inter-protein residue-residue interactions (RRIs), can be utilized to predict EEIs.

**Results:**

We evaluate the PPIIP methods for the RRI- and EEI-prediction tasks using all known experimentally determined 3D structures of human protein heterodimers from the Protein Data Bank available at the time of data collection. From these heterodimers, we determined ∼230 000 RRIs and ∼20 400 EEIs as ground truth. We provide the first evidence of the adaptability of existing PPIIP methods to predict EEIs, with a performance score of up to ∼76% based on the area under the receiver operating characteristic curve. Insights, data, and computational pipelines from our study can guide future developments of computational methods for solving the task of predicting EEIs.

**Availability and implementation:**

Data and source code are available at https://github.com/lieboldj/EEIpred.

## 1 Introduction

### 1.1 Background and motivation

Proteins physically interact with each other through protein-protein interactions (PPIs) to perform cellular functions. PPIs change with varying cellular conditions induced via several biological phenomena, such as alternative splicing (AS) (Yang *et al.* 2016). AS produces multiple protein isoforms by joining the protein-coding regions (i.e. exons) of a gene in different combinations (Wright *et al.* 2022). Abnormal protein isoforms have been shown to adversely affect human health (Climente-González *et al.* 2017, Zhang *et al.* 2021, Nikom and Zheng 2023), and thus studying AS-related PPI changes could reveal the molecular mechanisms of such effects. By the term “AS-related PPI change” we mean any two genes forming a PPI based on one of their protein forms, but not forming a PPI when alternate protein isoforms are present for one or both genes. For example, a protein form of the gene BAD is involved in a PPI with the longer protein isoform (Bcl-xL), but not with the shorter protein isoform (Bcl-xS), of the gene BCL2L1 (Yang *et al.* 2016). Similarly, much other experimental evidence also exists (Goode *et al.* 2000, Panda *et al.* 2003, Levy *et al.* 2005, Chung *et al.* 2015, Yang *et al.* 2016).

Using wet lab experiments to study PPI changes is expensive and time-consuming (Shoemaker and Panchenko 2007). Hence, computational methods to study AS-related PPI changes were developed (Ghadie *et al.* 2017, Narykov *et al.* 2021, Louadi *et al.* 2021). These methods rely on existing knowledge about protein domain-domain interactions (DDIs) as follows: they first integrate existing knowledge about DDIs to create a global DDI-resolved PPI network (i.e. a PPI network with information about which domains of the two proteins interact) and then use the global DDI-resolved PPI network to predict whether a PPI disappears if any of the two participating proteins lose the PPI-specific domain(s) due to AS (Ghadie *et al.* 2017). One such method is DIGGER (Louadi *et al.* 2021) which not only created a DDI-resolved PPI network but also delved deep into characterizing an exon-level view of PPIs, based on the following assumption: Because many AS events remove or add exons to form protein isoforms, looking at inter-protein exon-exon interactions (EEIs) may allow to explore AS-related PPI changes. DIGGER’s exon-level view of PPIs has been subsequently used to predict AS-related PPI changes and gain the underlying mechanistic understanding (Louadi *et al.* 2021, Gjerga *et al.* 2023).

Existing AS-related PPI prediction methods rely on EEI knowledge, which, although highly reliable, covers <0.5% of all known human PPIs (Berman *et al.* 2000, Oughtred *et al.* 2021). To fully capture the PPI network-based functional effects of AS, there is a need to extend the current limited knowledge of EEIs to all known human PPIs.

One way to do this is to predict novel EEIs using existing PPI interface prediction (PPIIP) methods (Esmaielbeiki *et al.* 2015), which can be grouped into two categories. The first category takes a single protein as input and predicts all possible PPI interfaces of the protein (Zeng *et al.* 2020, Sun and Frishman 2021, Tang *et al.* 2021, Yuan *et al.* 2021). It is not straight-forward to apply the methods from this category for the EEI prediction task. This is because even if one could modify such methods to predict PPI interfacing exons of a protein, these methods provide no knowledge about the exons of the protein’s PPI partners the predicted interfacing exons interact with. The second category of PPIIP methods, more directly applicable for EEI predictions, takes two proteins of a PPI (but lacking the corresponding interface information) as input and predicts which parts of the two proteins form the interface (Xue *et al.* 2015). Henceforth, whenever we use the term “PPIIP method,” we mean the PPIIP method from the second category. Typically, a PPIIP method uses ground truth knowledge about interacting (or non-interacting) residue pairs of PPI interfaces and protein features of residues to train a supervised machine learning model. Given any two test proteins $p_{1}$ and $p_{2}$ that are known to form a PPI but for which we lack the corresponding interface knowledge, the trained model predicts the likelihood of any two residues, $r_{1}$ from protein $p_{1}$ and $r_{2}$ from protein $p_{2}$, to interact, i.e. to form a residue-residue interaction (RRI) (Xue *et al.* 2015).

Because proteins interact in their 3D structural forms, utilizing 3D protein structures, in comparison to protein sequences, can more accurately predict the PPI interface regions. Motivated by this, several deep learning-based PPIIP methods have been developed that utilize 3D structures of proteins to predict RRIs (Dai and Bailey-Kellogg 2021, Sverrisson *et al.* 2021, Guo *et al.* 2022, Xie and Xu 2022, Lin *et al.* 2023a, 2023b, 2023c, Yuan *et al.* 2023, Rao *et al.* 2024, Si and Yan 2024). Note that rather than using the above PPIIP methods to predict EEIs, one could use docking (Meng *et al.* 2011), which aims to find an optimal spatial arrangement of any two 3D structures of proteins based on some predefined optimization criteria, e.g. minimizing the average distance of every residue pair between the two proteins. However, docking has high computational costs, taking a few minutes to several hours or even days to process one protein pair (Meng *et al.* 2011, Bender *et al.* 2021). Given ∼1 000 000 known human PPIs in BioGRID (Stark *et al.* 2006), using docking to predict EEIs over the entire known human PPIs would need at least ∼2 years if one protein pair per minute is processed. Similar to docking, the latest developments in protein complex predictions, such as AlphaFold (AF)-multimer (Evans *et al.* 2021) or the Kiharalab method from the 16th Critical Assessment of Structure Prediction (Kagaya *et al.* 2026), which take protein sequences as input and predict the 3D structure of the corresponding protein complex, typically take more than an hour to process one protein pair (Bryant *et al.* 2022) (Section 3.8). In contrast, the existing 3D structure-based PPIIP methods (mentioned above) have been specifically designed to predict PPI interfaces and are typically much faster, with an average runtime of only a few seconds to predict interfacing residues of a protein pair (Dai and Bailey-Kellogg 2021, Sverrisson *et al.* 2021, Yuan *et al.* 2023). However, it has not been evaluated whether, or how well, such existing PPIIP methods can be used for the EEI prediction task.

### 1.2 Our contributions

**Figure 1:**
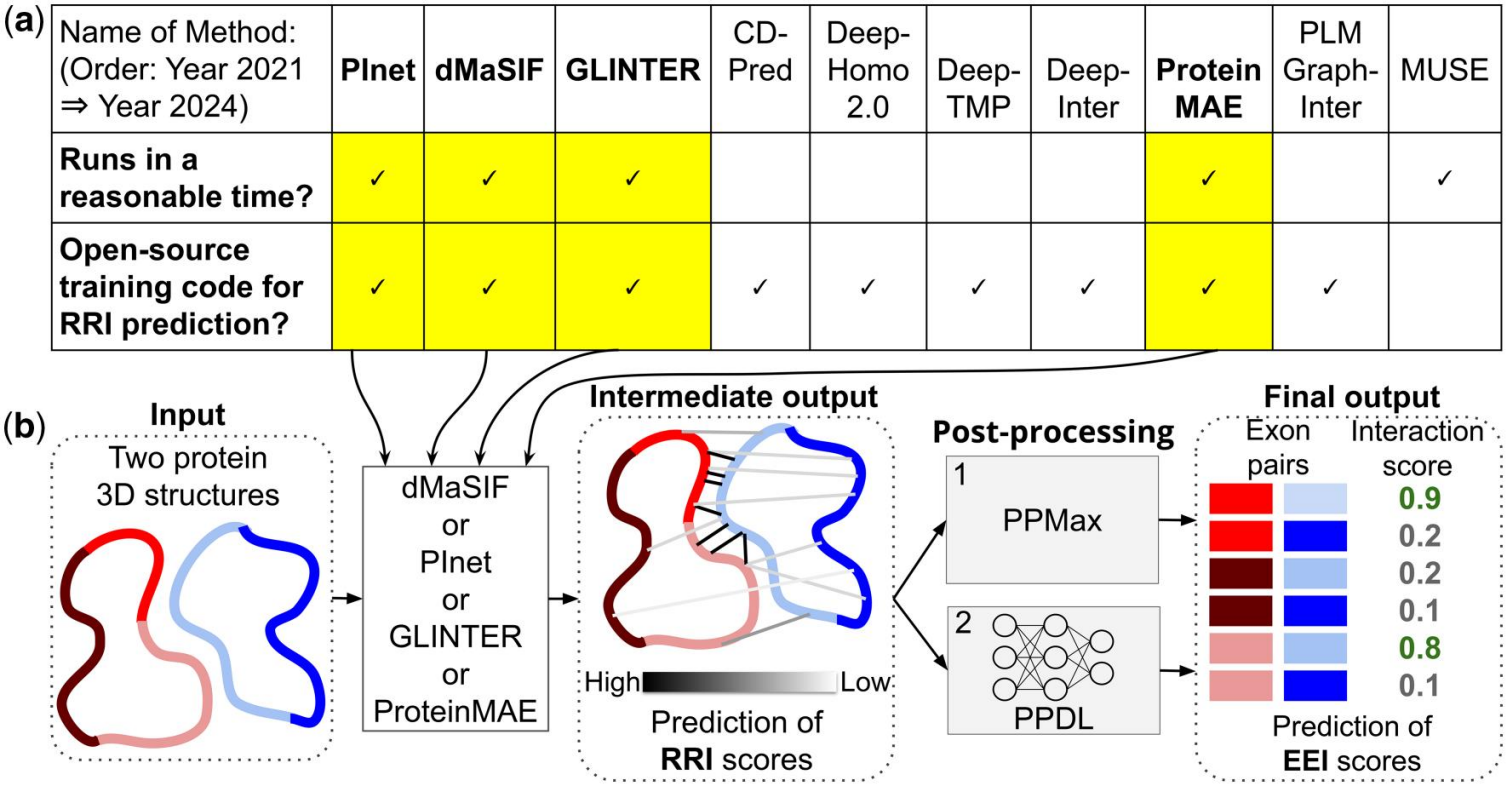
The computational pipeline of our study. Panel (a) names of the 10 PPIIP methods (Dai and Bailey-Kellogg 2021, Sverrisson *et al*. 2021, Xie and Xu 2022, Guo *et al*. 2022, Lin *et al*. 2023a, 2023b, 2023c, Yuan *et al*. 2023, Si and Yan 2024, Rao *et al*. 2024) (top row) that we consider, along with the two requirements used for their further consideration (leftmost column). A tick mark within a cell denotes that the method fulfills the corresponding requirement. Panel (b) given the 3D structures of two proteins (represented as closed loops) with exon annotations (highlighted in different shades within a protein) as input, we first apply a PPIIP method (either dMaSIF, PInet, GLINTER, or ProteinMAE) to obtain RRI scores (“Intermediate output”). Given the RRI scores, we then obtain the EEI scores (“Final output”) using two post-processing approaches, i.e. PPMax (post-processing by taking the maximum of RRI scores of an exon pair) and PPDL (post-processing by learning patterns of RRI scores of exon pairs via deep learning). See Section 2 for details.

In this study, we focus on predicting novel EEIs within known PPIs. Such novel EEI knowledge could then be used by existing computational tools, e.g. DIGGER or NEASE, that rely on EEIs to capture the effect of AS on PPI perturbations. To predict novel EEIs, we consider 10 PPIIP methods that were published between the years 2021 and 2024, as per our literature search in Google Scholar. Of the 10 PPIIP methods, we could only consider those that (i) are open-source allowing to train a model using new data and that (ii) run in a reasonable time (see Section 2.2 for details). Four PPIIP methods fulfill these two requirements: Differentiable Molecular Surface Interaction Fingerprinting (dMaSIF) (Sverrisson *et al.* 2021), Protein Interface Network (PInet) (Dai and Bailey-Kellogg 2021), Graph Learning of Inter-protein contacts (GLINTER) (Xie and Xu 2022), and a Protein Masked AutoEncoder (ProteinMAE) (Yuan *et al.* 2023). We evaluate the four methods in the RRI and EEI prediction tasks (Fig. 1). To do this, we use all reviewed UniProt human proteins available at the time of data collection which map to ∼13 000 high-confidence co-resolved 3D structures of heterodimeric protein pairs from the Protein Data Bank (PDB) (Berman *et al.* 2000). Because there is no ground truth EEI data available, we create such data using three existing methods that detect PPI interfaces from co-resolved proteins (Newaz *et al.* 2024), resulting in three datasets with a total of ∼20 400 EEIs encompassing ∼230 000 RRIs (Section 2.1).

Given the considered four PPIIP methods and the three datasets, we first evaluate the methods on the RRI prediction task. We do this because the four methods have never been compared to each other for RRI prediction. This allows us to benchmark their performance within their designated scope. Additionally, this helps us to better interpret their performance on the proposed EEI prediction task, which we perform next. For a robust performance evaluation, given a dataset, we divide it into five subsets of protein pairs such that there is <30% sequence identity for any two proteins across subsets. Given a subset, we use it as an independent test data, while we divide the remaining four subsets as training (three subsets) and validation (one subset) data (Sections 2.1 and 2.4). We evaluate the performance of each method based on the area under the receiver operating characteristic (AU-ROC) curve, the area under the precision-recall curve (AU-PRC), as well as Matthew’s correlation coefficient (MCC), precision, recall, and F-score for a certain false discovery rate (FDR).

For both RRI and EEI prediction tasks, while the results vary with the choice of the dataset, each method performs significantly better than random with AU-ROC values of the best method(s) reaching up to ∼89% and ∼76% for the RRI and EEI prediction tasks, respectively. In particular, for the EEI prediction task, the MCC, F-score, precision, and recall reach up to ∼31%, ∼50%, ∼96%, and ∼38%, respectively, with an FDR ≤5%. To demonstrate the applicability of our approach, we first show that prediction performance is nearly identical when using either experimental (PDB) or predicted (AF) structures. Accordingly, we apply the best performing EEI prediction model to predict novel EEIs for a curated set of 261 high-confidence human PPIs for which no EEIs are known. We predict (with 0% FDR) 436 novel EEIs covering 111 PPIs. These novel EEIs could be candidates for future experimental validation. Our study provides, for the first time, insights, data, and computational pipelines for the EEI prediction task. These can guide future developments of 3D protein structure-based computational methods for the EEI prediction task.

## 2 Data and methods

### 2.1 Dataset curation

We consider all 2349 PDB entries (downloaded in January 2022) which (i) contain at least one pair of co-resolved proteins (determined either by X-ray crystallography or electron microscopy, or by nuclear magnetic resonance) among all 20 378 reviewed human proteins from UniProt (Uniprot Consortium 2019) (downloaded in January 2022) and (ii) have sufficient 3D structural resolution if the structure was detected by X-ray crystallography or electron microscopy (≤3 Å). The above steps result in 13 967 unique co-resolved protein pairs among 2882 unique proteins. We map exon information onto each of the 2882 UniProt protein sequences using the Ensembl database (Martin *et al.* 2023), as follows.

**Figure 2:**
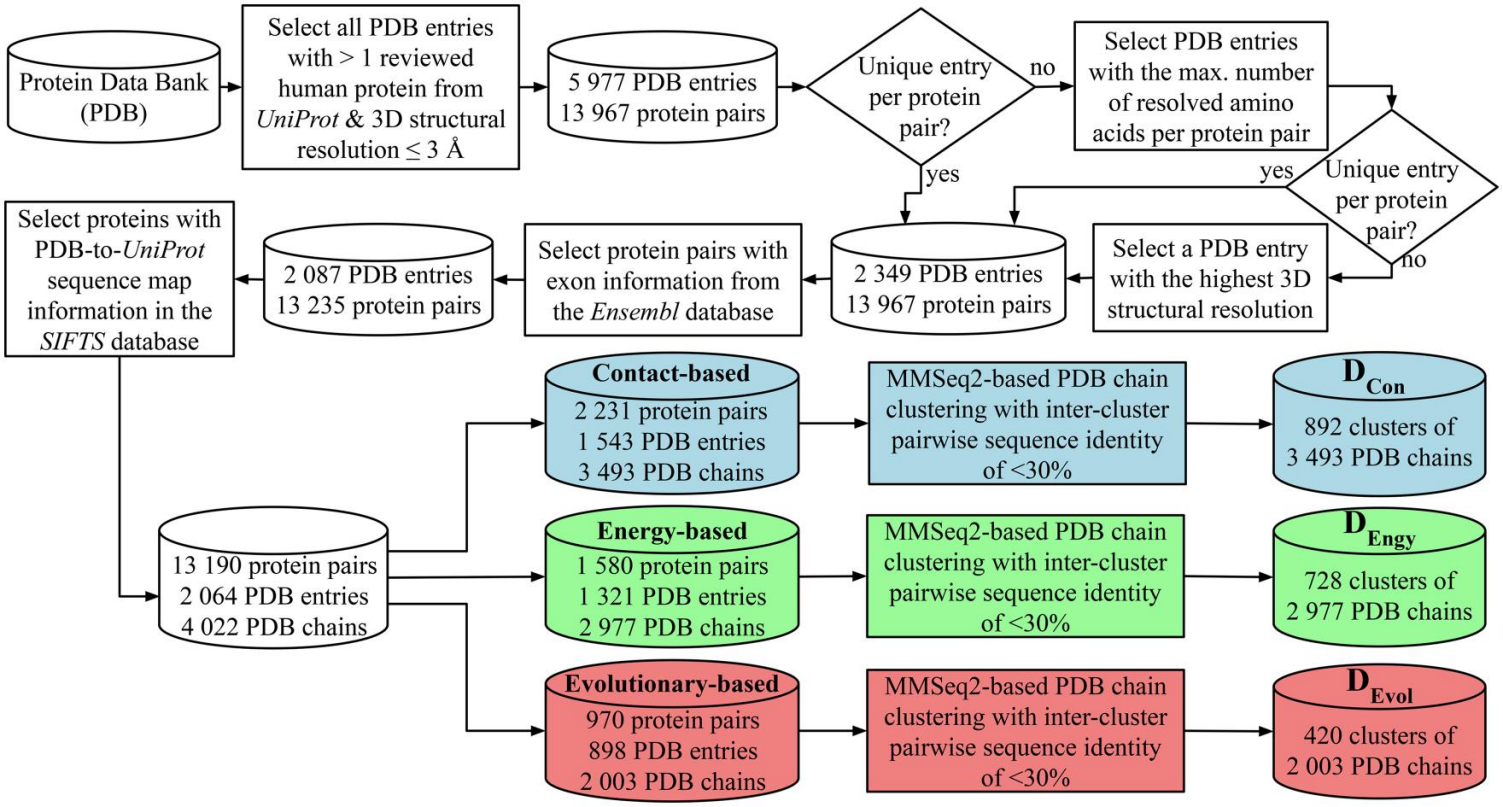
Overview of our data generation pipeline. We use all available reliable PDB entries to create three datasets based on three EEI detection approaches, i.e. contact-based, energy-based, and evolution-based. Then, we apply MMSeqs2 on each of the three datasets to derive the corresponding sets of sequence non-redundant clusters of PDB chains, i.e. $D_{\text{Con}}$, $D_{\text{Engy}}$, and $D_{\text{Evol}}$ (see Section 1.1, available as supplementary data at *Bioinformatics Advances* online, for details).

We use the BiomaRt package (Durinck *et al.* 2005) to map UniProt IDs to the Ensembl transcripts. Because Ensembl has information for more than one transcript per gene, there is no one-to-one mapping between a UniProt protein sequence and the Ensembl transcripts. To select a unique transcript per protein, we retain the Ensembl transcript that aligns entirely (i.e. with no gaps and no mismatches) with the given protein sequence based on the Needleman-Wunsch pairwise global sequence alignment. If we do not find any such transcript for any protein, we exclude the protein from our study. For every protein with a mapped transcript, we map their amino acid sequence positions onto their 3D resolved structures from PDB using the Structure integration with function, taxonomy, and sequence (SIFTS) database (Velankar *et al.* 2013, Dana *et al.* 2019). After successful mapping of data across different databases, 2064 PDB entries (4022 PDB chains) constituting 13 190 unique co-resolved protein pairs among 2649 proteins remain (Fig. 2 and Sections 1.1.1–1.1.3, available as supplementary data at *Bioinformatics Advances* online).

Given a pair of co-resolved proteins $p_{1}$ and $p_{2}$, using established criteria (Faisal *et al.* 2017, Newaz *et al.* 2020, 2022, Xie and Xu 2022), we define an RRI between residues $r_{1}$ from $p_{1}$ and $r_{2}$ from $p_{2}$, if and only if the 3D Euclidean distance between any of the heavy atoms (i.e. carbon, nitrogen, oxygen, and sulphur) of $r_{1}$ is within 6 Å to any of the heavy atoms of $r_{2}$.

For each pair of exons $e_{1}$ from $p_{1}$ and $e_{2}$ from $p_{2}$, we define two exons to form an EEI using three approaches based on Euclidean distance between residues, thermodynamic stability of the interface, and evolutionary conservation of the interface, leading to three datasets. First, to form our *contact-based* dataset, we define an EEI between two exons $e_{1}$ and $e_{2}$ if and only if at least one residue of $e_{1}$ forms an RRI (as defined above with a distance cut off of 6 Å) with at least one residue of $e_{2}$. Second, to form our *energy-based* dataset, we use a thermodynamics-based method called Protein Interfaces, Surfaces, and Assemblies (PISA) (Krissinel and Henrick 2007) to identify biologically relevant interfaces. PISA first computes a solvation free energy gain of an interface, which quantifies the thermodynamic changes that occur due to the interface formation. Then it computes the probability of observing the same solvation free energy gain by chance when a random set of atoms (with the same area as that of the interface) is picked from the non-interfacing surfaces of the two proteins. PISA labels an interface as biologically relevant if the corresponding probability is <.5. We define an EEI between $e_{1}$ and $e_{2}$ if and only if they overlap with the identified interfaces. Third, to form our *evolutionary-based* dataset, we use an evolution-based method called Evolutionary Protein-Protein Interface Classifier (EPPIC) (Bliven *et al.* 2018) to identify biologically relevant interfaces. EPPIC characterizes an interface as biologically relevant if the interface surface area is more than 2200 Å^2^, while it characterizes an interface to be an artifact of crystallization if the interface surface area is <400 Å^2^. For interface surface areas between 400 Å^2^ and 2 200 Å^2^, EPPIC uses information about the evolutionary conservation of the corresponding protein sequences to characterize whether the interface is biologically relevant or not. For our study, we run EPPIC using default parameters. We define an EEI between $e_{1}$ and $e_{2}$ if and only if they overlap with the identified interfaces.

For each of the three datasets, we only consider those PDB entries that (i) contain at least one interacting exon pair and (ii) could be pre-processed by each of the four considered PPIIP methods (Section 2.2). To avoid sequence leakage across training and test sets, for each dataset, we cluster PDB chains at <30% identity using MMSeqs2 (Steinegger and Söding 2017), resulting in 892, 728, and 420 non-redundant clusters of PDB chains (i.e. protein sequences) for the contact-based ($D_{\text{Con}}$), energy-based ($D_{\text{Engy}}$), and evolutionary-based ($D_{\text{Evol}}$) datasets, respectively (Section 1.1.5, available as supplementary data at *Bioinformatics Advances* online).

### 2.2 PPI interface prediction methods

Among the 10 considered PPIIP methods (Table 1, available as supplementary data at *Bioinformatics Advances* online), we could only use four, i.e. dMaSIF, GLINTER, PInet, and ProteinMAE, that provide open-source program code to train a model and have reasonable runtime (Section 1.2, available as supplementary data at *Bioinformatics Advances* online). The four methods use different pre-processing of input proteins, initial protein features, and deep learning architectures. In terms of pre-processing of proteins, while dMaSIF, PInet, and ProteinMAE model 3D structures of proteins as point clouds albeit using different approaches, GLINTER relies on protein contact maps. In terms of initial protein features, while dMaSIF, PInet, and ProteinMAE rely on the geometric and physicochemical features of proteins, GLINTER additionally includes evolutionary information relying on sequence homology. In terms of the deep learning architecture, while dMaSIF, PInet, and ProteinMAE use geometric deep learning with differences in their underlying architectures, GLINTER uses graph-based learning and attention-based models. We use each of the four methods to predict RRI scores for each pair ($r_{1}$, $r_{2}$) of residues between two input proteins $p_{1}$ and $p_{2}$, where $r_{1}$ is from $p_{1}$ and $r_{2}$ is from $p_{2}$, as follows: dMaSIF and ProteinMAE output interaction scores of atom pairs across the two proteins. Here, we compute an RRI score as the maximum of all atom pair scores of two residues. PInet outputs the probability of a residue to be part of an interface. Here, we compute an RRI score as the product of the individual probability scores of two residues. GLINTER directly outputs a likelihood of a residue pair to interact, which we use as the corresponding RRI score. A higher RRI score indicates a higher likelihood of interaction between the corresponding residue pair. See Sections 1.2.1–1.2.4, available as supplementary data at *Bioinformatics Advances* online, for details.

**Table 1:** Test set sizes of $D_{\text{Con}}$, $D_{\text{Engy}}$, and $D_{\text{Evol}}$, and their overlaps^a^.

	Number of		
Dataset(s)	Protein pairs	Interacting exon pairs	Non-interacting exon pairs
$D_{\text{Con}}$ only	381	1965	8093
$D_{\text{Engy}}$ only	216	2047	3019
$D_{\text{Evol}}$ only	126	1364	2403
$D_{\text{Con}}\cap D_{\text{Engy}}$	63	297	608
$D_{\text{Con}}\cap D_{\text{Evol}}$	25	123	376
$D_{\text{Engy}}\cap D_{\text{Evol}}$	22	495	598
$D_{\text{Con}}\cap D_{\text{Evol}}\cap D_{\text{Engy}}$	35	273	475

a The symbol ∩ denotes intersection between the test sets.

### 2.3 Post-processing approaches for EEI prediction

Given two proteins $p_{1}$ and $p_{2}$ the corresponding RRI predictions from a PPIIP method, we propose two post-processing approaches to predict the EEI scores between any two exons $e_{1}$ from $p_{1}$ and $e_{2}$ from $p_{2}$. The first post-processing approach (referred to as post-processing via the maximum RRI score or PPMax for short) calculates the score for an exon pair ($e_{1}$,$e_{2}$) as the maximum predicted RRI score among all residue pairs ($r_{1}$,$r_{2}$), where $r_{1}$ is a residue in exon $e_{1}$ and $r_{2}$ is a residue in exon $e_{2}$. The second post-processing approach (referred to as post-processing via deep learning or PPDL for short) is based on a convolutional neural network (CNN) (Schmidhuber 2015) where, given a set of interacting and non-interacting exon pairs, it first trains a model to learn distinguishing patterns of the predicted RRI scores for interacting versus non-interacting exons. Then, given two test exon pairs, it predicts the corresponding EEI score (see Section 1.3, available as supplementary data at *Bioinformatics Advances* online, for more details on PPDL and Fig. 1, available as supplementary data at *Bioinformatics Advances* online, for our PPDL architecture).

### 2.4 Model training and evaluation

For each of the three sequence non-redundant PDB chain clusters, i.e. $D_{\text{Con}}$, $D_{\text{Engy}}$, and $D_{\text{Evol}}$, we aim to train and test a machine learning model similar to five-fold cross-validation. To do this, for each of $D_{\text{Con}}$, $D_{\text{Engy}}$, and $D_{\text{Evol}}$, we first split the corresponding clusters into five non-overlapping subsets (or folds) using a round-robin forward-backward assignment, in order to keep the number of PDB chains across the five subsets as similar as possible (Section 1.4, available as supplementary data at *Bioinformatics Advances* online). We use each subset as an independent test set (henceforth, referred to simply as “test set” for brevity) and the remaining four subsets for validation (one subset) and training (three subsets). This yields 60 RRI models (3 datasets×4 PPIIP methods×5 folds) and 120 EEI models (3 datasets×4 PPIIP methods×5 folds×2 post-processing methods). Note that our EEI prediction models predict interaction scores between pairs of exons across proteins, while the RRI prediction models predict interaction scores between pairs of residues across proteins. For both categories of models, no explicit 3D structures of the corresponding protein complexes are predicted. We evaluate each model using six performance measures: AU-ROC, AU-PRC, MCC, precision, recall, and F-score. For threshold-dependent measures (i.e. MCC, precision, recall, and F-score), we set decision thresholds by controlling FDRs at ≤1%, 2%, 3%, 4%, and 5% on non-interactions in the training data (Section 1.5 and Fig. 2, available as supplementary data at *Bioinformatics Advances* online).

## 3 Results and discussion

We evaluate four state-of-the-art 3D structure-based PPIIP methods, i.e. dMaSIF, PInet, GLINTER, and ProteinMAE, on how well the methods can predict the PPI interface at the exon-level (i.e. EEIs) between two proteins. Because we generate the ground truth EEI datasets for the first time, to have more insights, we analyze their overlaps and structural characteristics (Section 3.1). The considered PPIIP methods were, to the best of our knowledge, never compared to each other on their original task of predicting the PPI interface at the residue-level (i.e. RRIs). Hence, we compare them on the RRI prediction task (Section 3.2). To adapt the PPIIP methods for the EEI prediction task, we propose two post-processing approaches, which we compare against each other to select the best one for further analyses (Section 3.3). Using the best post-processing approach, we use the PPIIP methods to evaluate how well they can predict EEIs (Section 3.4). Then, we compare the PPIIP methods on how well they predict PPI interfaces across two different levels, i.e. residue-level versus exon-level (Section 3.5). To assess the applicability to predicted structures, we compare the performance on experimental versus predicted protein structures (Section 3.6). Next, we apply the best performing PPIIP method to known PPIs lacking EEI annotations to generate a list of predicted EEIs and discuss two concrete examples (Section 3.7). Finally, we evaluate the runtime of the PPIIP methods (Section 3.8).

### 3.1 Dataset overview and structural characteristics

**Figure 3:**
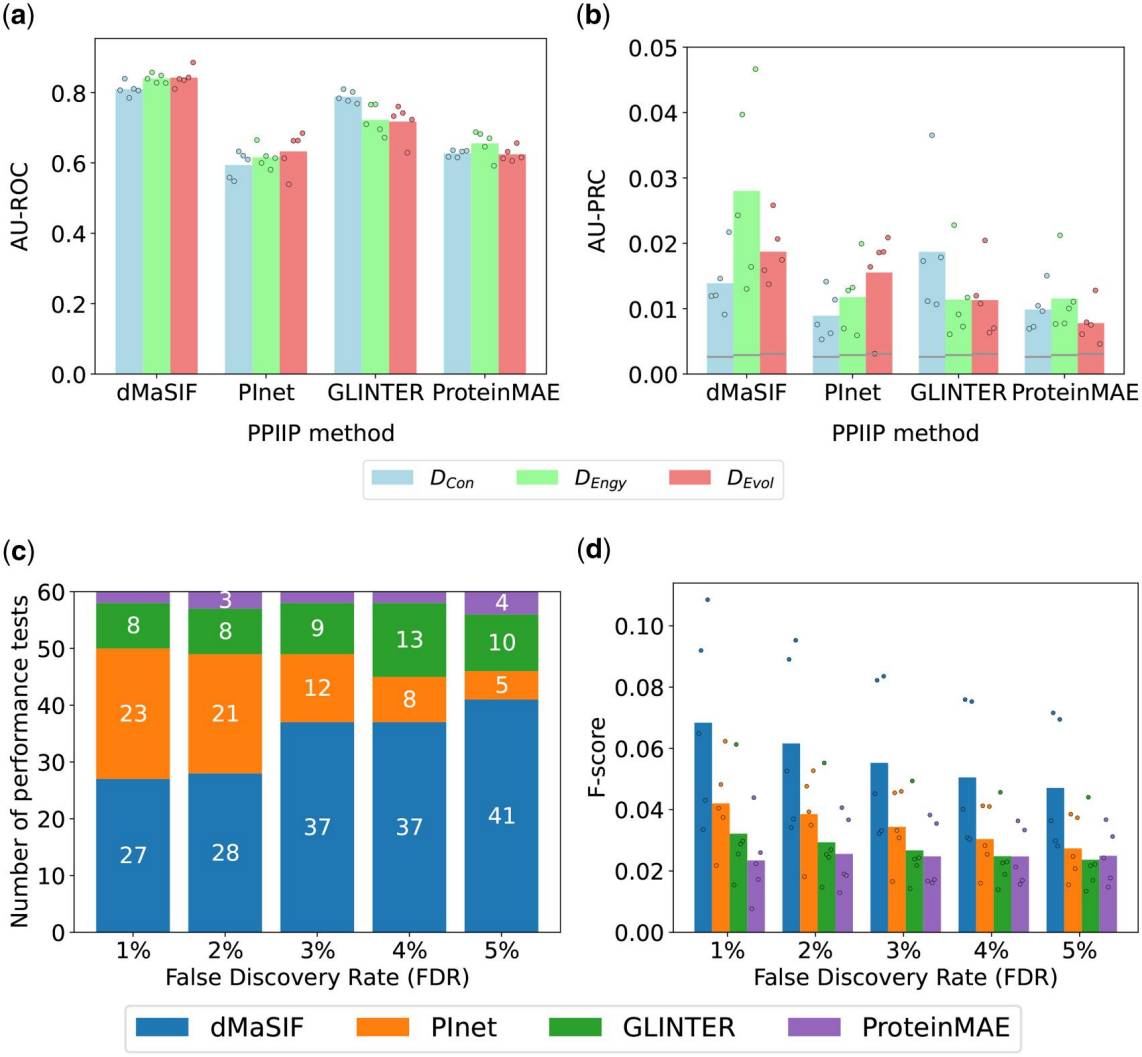
Performance of the PPIIP methods on the RRI prediction task. Panels (a and b) show mean AU-ROC and AU-PRC, respectively, across 5 folds for each dataset (colors), with points for individual folds. In panel (b), gray lines mark the fraction of interacting residues. Panel (c) shows, for each FDR on the x-axis, the number of performance tests (y-axis) in which each method ranks best (indicated by different colored compartments of the bar and the corresponding numbers within those compartments). Panel (d) shows the F-scores of the PPIIP methods for the $D_{\text{Engy}}$ dataset with individual data points indicate the value for each test set. See Fig. 7, available as supplementary data at *Bioinformatics Advances* online, for precision-recall curves. The above results are for the RRI definition based on a 6 Å distance cutoff (Section 2.1, available as supplementary data at *Bioinformatics Advances* online). We find similar results for alternative RRI definitions based on distance cutoffs of 4 Å (Table 2, available as supplementary data at *Bioinformatics Advances* online—4Å) and 8 Å (Table 2, available as supplementary data at *Bioinformatics Advances* online—8Å).

**Figure 4:**
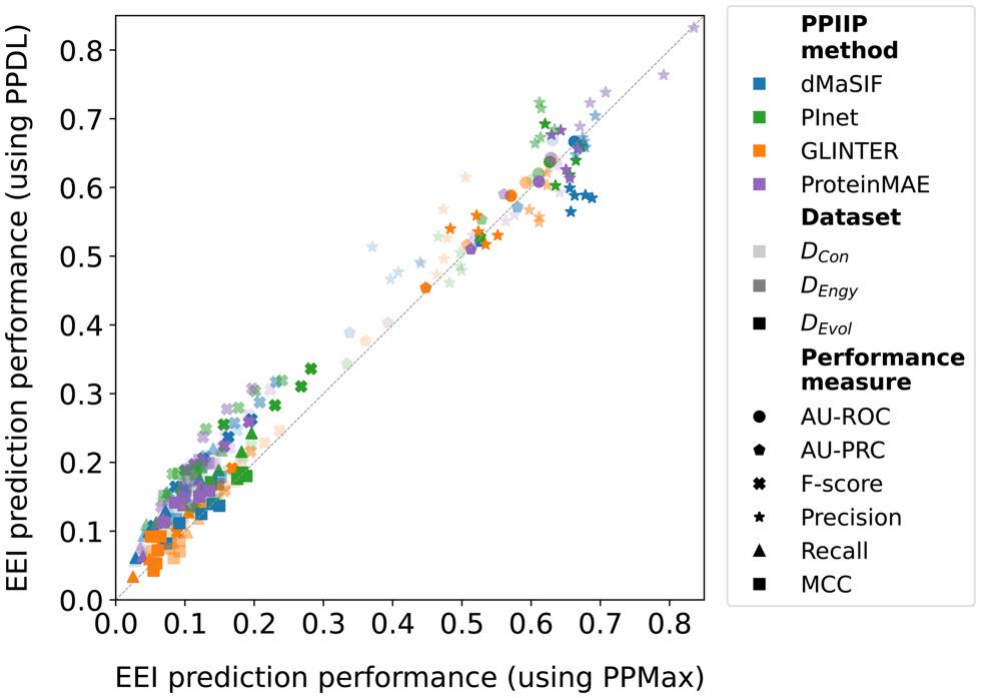
Performance of the PPIIP methods on the EEI prediction task using the PPMax (x-axis) versus the PPDL (y-axis) post-processing approaches. Each point in the plot represents the average (over the five test sets) performance of a PPIIP method using PPDL versus using PPMax for a given combination of dataset, performance measure, and FDR choice (if applicable). For detailed results, see Tables 5 and 6, available as supplementary data at *Bioinformatics Advances* online.

**Figure 5:**
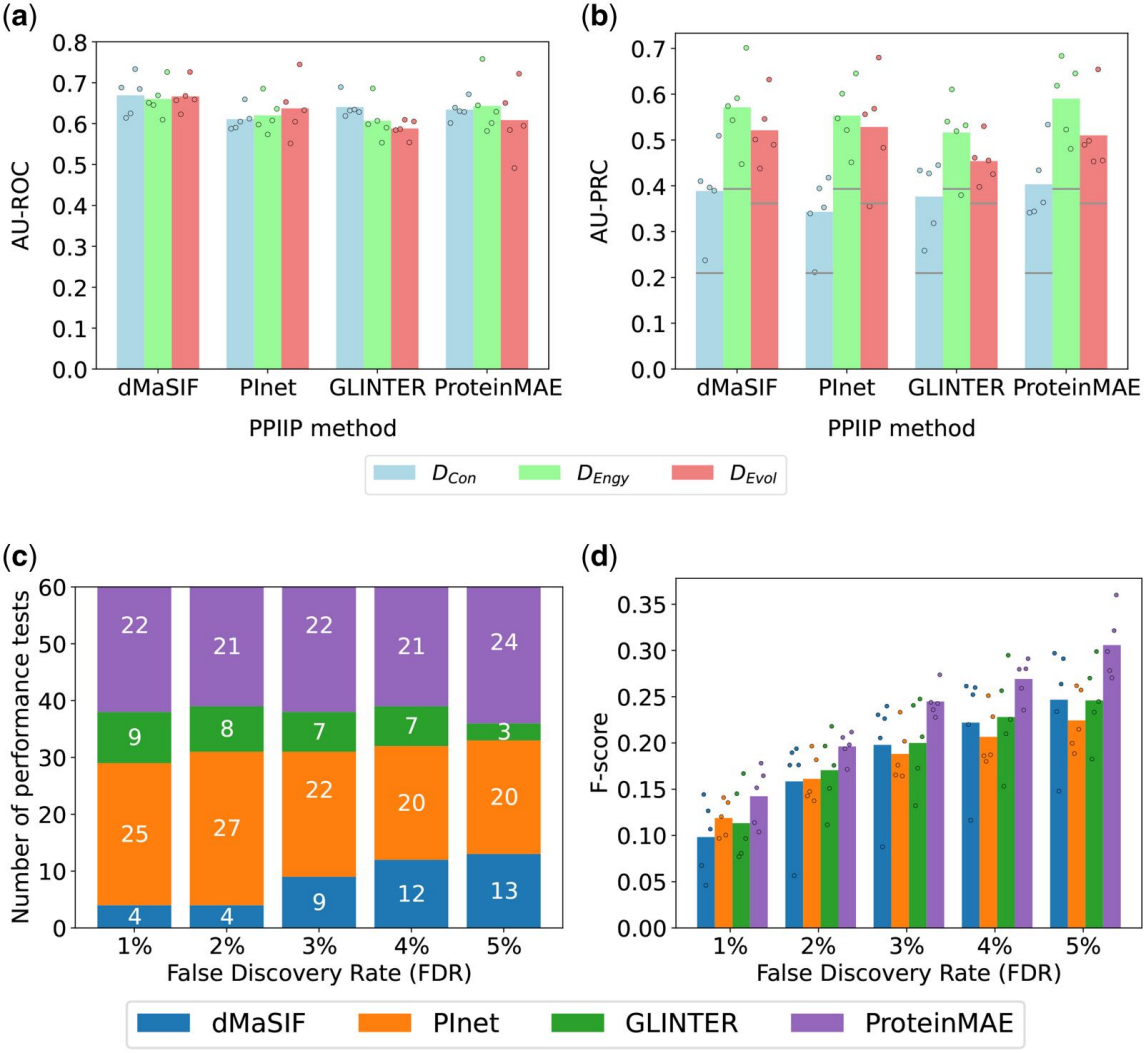
Performances of the PPIIP methods on the EEI prediction task. (a–d) Mirror Fig. 3 for AU-ROC, AU-PRC (gray lines mark interacting-exon fractions), top-rank counts, and F-scores on $D_{\text{Con}}$, respectively. See Fig. 10, available as supplementary data at *Bioinformatics Advances* online, for precision-recall curves.

**Figure 6:**
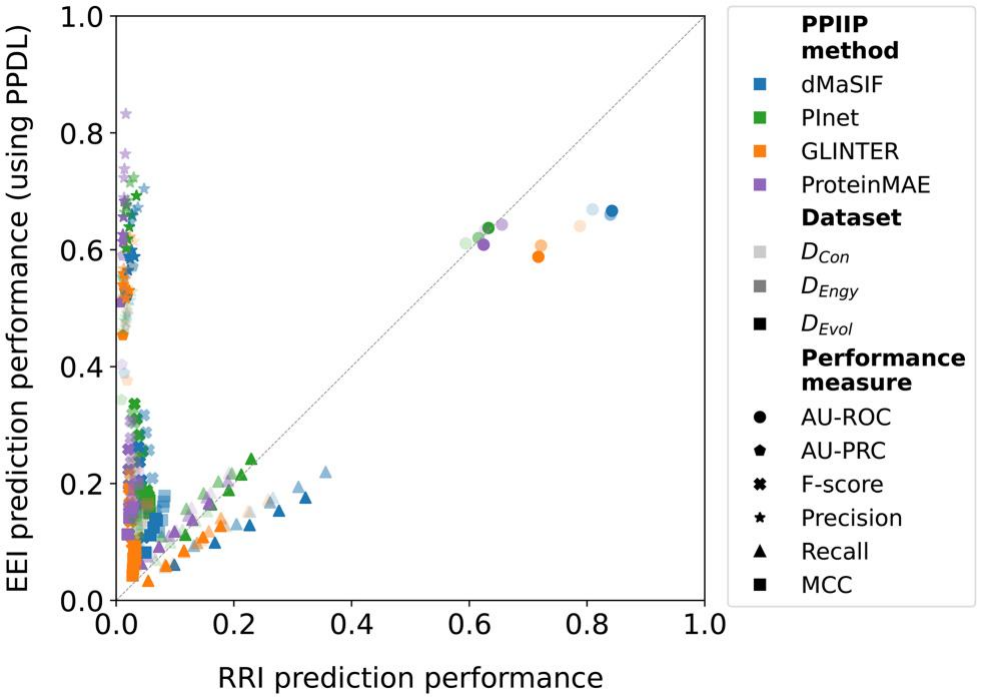
Performances of the PPIIP methods on the RRI (x-axis) versus the EEI (y-axis) prediction tasks. Each point in the plot represents the average (over the five test sets per dataset) performance of a PPIIP method using PPDL versus RRI prediction for a given combination of dataset, performance measure, and FDR choice (if applicable).

For each of $D_{\text{Con}}$, $D_{\text{Engy}}$, and $D_{\text{Evol}}$, we define the set of protein pairs as the union across the five test sets (Section 2.4). We then quantify overlaps at the protein and exon-pair levels and compare interacting and non-interacting exon pairs by secondary structure and intrinsically disordered region (IDR) content across datasets (Section 1.6, available as supplementary data at *Bioinformatics Advances* online). The datasets show marginal overlaps, with $D_{\text{Con}}$ having the most protein pairs and $D_{\text{Engy}}$ having the most exon pairs (Table 1). For each dataset, we find significant (*q*-value <.05) differences between interacting and non-interacting exon pairs for at least one secondary structural label (either α-helix, β-sheet, or coil-turn). Regarding IDR content, we find significant differences for $D_{\text{Con}}$ and $D_{\text{Engy}}$ (but not for $D_{\text{Evol}}$), with interacting exon pairs having more IDR content than non-interacting exon pairs. For detailed results, see Section 1.7.1 and Figs. 3–6, available as supplementary data at *Bioinformatics Advances* online. These differences could also percolate into the performance differences of a PPIIP method across $D_{\text{Con}}$, $D_{\text{Engy}}$, and $D_{\text{Evol}}$.

### 3.2 Evaluation of the PPIIP methods on the RRI prediction task

For each of $D_{\text{Evol}}$, $D_{\text{Engy}}$, and $D_{\text{Con}}$, every PPIIP method performs better than random, achieving AU-ROC values (averaged over the five test sets of a dataset) ranging from 0.59 to 0.84 (Fig. 3a and Table 2, available as supplementary data at *Bioinformatics Advances* online). dMaSIF consistently performs best, with AU-ROC values ranging (across datasets) from 0.81 to 0.84. The performance of GLINTER follows dMaSIF, with AU-ROC values ranging from 0.72 to 0.79. PInet and ProteinMAE perform worse than dMaSIF and GLINTER across all datasets. Due to the imbalance in RRI datasets with ∼350× more negative than positive RRIs (Table 3, available as supplementary data at *Bioinformatics Advances* online), AU-ROC is less likely to give an accurate estimate of a method’s performance than AU-PRC (Davis and Goadrich 2006). By this, we mean that unlike AU-ROC, which can be insensitive to data imbalance, AU-PRC focuses specifically on quantifying performance based on positive labels, making it a more sensitive and informative metric in highly imbalanced datasets with few positive data points (here, positive RRIs). Note that this data imbalance is drastically reduced (∼1:4) at the exon level (Table 3, available as supplementary data at *Bioinformatics Advances* online). We find that AU-PRC values are below 0.05 for each of the PPIIP methods for any dataset (Fig. 3b). The low AU-PRC values indicate that the underlying problem of RRI predictions is far from being solved and novel improved PPIIP methods are required.

**Table 2:** Computational runtimes of PPIIP methods and AF models.

Method	Average pre-processing time (s)	Average processing time (s)	Total time (s) (set of 10 protein pairs)
dMaSIF	2	3	46
PInet	36	1	369
GLINTER	359	1	3599
ProteinMAE	2	2	37
AF-Multimer	5334	1231	65 643
AF 3	1221	88	13 088

In addition to AU-ROC and AU-PRC, we measure the methods’ performances using MCC, precision, recall, and F-score at a prediction score decision threshold that allows for an FDR of t% (Section 2.4). Given a dataset and a PPIIP method, for each FDR choice, we perform 60 performance tests based on the combination of the four performance measures (i.e. MCC, precision, recall, and F-score), the three datasets, and the five test sets. When we rank (from best to worst) the PPIIP methods based on the total number of performance tests in which they perform best, we find that dMaSIF is always ranked first and ProteinMAE is always ranked fourth for each FDR choice, while PInet and GLINTER switch between ranks 2 and 3 depending on the FDR choice (Fig. 3c). Using paired Wilcoxon signed-rank tests with Benjamini–Hochberg correction between PPIIP methods, we find significantly (*q*-value <.05) better performance for dMaSIF across multiple comparisons (see Table 4a, available as supplementary data at *Bioinformatics Advances* online and Section 1.7.2, available as supplementary data at *Bioinformatics Advances* online, for more details).

For F-score, while dMaSIF performs best in the majority of FDR choices across $D_{\text{Engy}}$ and $D_{\text{Evol}}$, GLINTER consistently ranks highest for $D_{\text{Con}}$. Note that there is a decrease in the F-score values with the increasing FDR (Fig. 3d). This is because, as the FDR choice increases, although the recall values of the PPIIP method increase, their precision values decrease drastically (Table 2 and Fig. 8, available as supplementary data at *Bioinformatics Advances* online).

To summarize, ProteinMAE always performs worst irrespective of the choice of dataset or performance measure. Note that ProteinMAE was shown to outperform dMaSIF in its original study but for the task of predicting interfacing residues of a protein without considering the other protein partner. In contrast, in our study, we evaluate the task of predicting RRIs of two proteins that form a PPI. Hence, our results do not contradict the results of ProteinMAE’s original study. For some combinations of datasets and performance measures, GLINTER or PInet show better performance than dMaSIF, but in the majority of cases, dMaSIF performs best.

### 3.3 Comparison of PPDL and PPMax on the EEI prediction task

We compare the two post-processing approaches (Section 2.3), i.e. PPMax and PPDL, on the EEI prediction task. Given a PPIIP method, for a majority of the combinations of performance measures and datasets, PPDL outperforms PPMax (Fig. 4). Using paired Wilcoxon signed-rank tests with Benjamini–Hochberg correction (using 15 performance values based on the combination of the three datasets and the five test sets), we find significant (*q*-value <.05) differences between PPDL and PPMax performances for recall, F-score, and MCC, but not for AU-ROC and AU-PRC (see Section 1.7.3 and Fig. 9, available as supplementary data at *Bioinformatics Advances* online).

From a PPI interface point of view, interactions between exon pairs potentially rely on multiple (rather than single) RRIs along with their interaction patterns. However, the exact number of such RRIs or their patterns that define an EEI is not known. PPDL with its CNN learns such RRI patterns of EEIs, which likely results in its better performance than PPMax. This also signifies that future improvements on learning patterns from predicted RRIs scores have the potential to improve EEI prediction. Henceforth, we only show results for PPDL, while we provide the corresponding results for PPMax in the supplement.

### 3.4 Evaluation of the PPIIP methods on the EEI prediction task

For each of $D_{\text{Evol}}$, $D_{\text{Engy}}$, and $D_{\text{Con}}$, every PPIIP method performs non-randomly with AU-ROC values (averaged over the five test sets of a dataset) ranging from 0.59 to 0.67 (Fig. 5a and Table 6, available as supplementary data at *Bioinformatics Advances* online). dMaSIF consistently performs best, with AU-ROC values ranging (across datasets) between 0.66 and 0.67. The performance of ProteinMAE follows dMaSIF with AU-ROC values ranging from 0.61 to 0.64. PInet and GLINTER closely follow ProteinMAE with AU-ROC values ranging from 0.61 to 0.64 for PInet and from 0.59 to 0.64 for GLINTER. With respect to AU-PRC (averaged over the five test sets of a dataset), ProteinMAE and dMaSIF perform best with values ranging (across datasets) from 0.40 to 0.59 for ProteinMAE and from 0.39 to 0.57 for dMaSIF (Fig. 5b). Among PInet and GLINTER, PInet performs better than GLINTER with one exception: for the $D_{\text{Con}}$ dataset, GLINTER outperforms PInet.

Similar to evaluations of RRI predictions (Section 3.2), we perform 60 performance tests for each PPIIP method. When we rank (from best to worst) the PPIIP methods based on the total number of performance tests in which they perform best, we find that the first rank is always occupied by either ProteinMAE or PInet, with the top rank shared between the two for the FDR choice of 3%. Between dMaSIF and GLINTER, we find that dMaSIF is ranked better than GLINTER for three FDR choices (3%–5%), while the opposite is true for the remaining FDR choices of 1% and 2% (Fig. 5c). However, we do not find any statistically significant (*q*-value <.05) differences (Table 4b, available as supplementary data at *Bioinformatics Advances* online).

For F-score, we find that there is an increase as the FDR increases (Fig. 5d). This happens because, as the FDR increases, for each method, we see a larger increase in recall with a much smaller drop in precision (Table 6, available as supplementary data at *Bioinformatics Advances* online). For the $D_{\text{Con}}$ dataset, for each FDR choice, ProteinMAE performs best (Fig. 5d). For $D_{\text{Engy}}$ and $D_{\text{Evol}}$, PInet is best, followed by dMaSIF, ProteinMAE, and GLINTER. The only exception is at 1% FDR for $D_{\text{Evol}}$, where ProteinMAE and dMaSIF switch positions (Fig. 11, available as supplementary data at *Bioinformatics Advances* online).

To summarize, dMaSIF shows on average the best performance based on AU-ROC. However, with respect to other performance metrics, in the majority of cases, ProteinMAE and PInet show the best performance. GLINTER and dMaSIF perform the worst irrespective of the choice of dataset or threshold-dependent performance measure.

### 3.5 Comparison of the PPIIP methods on the EEI versus RRI prediction tasks

To evaluate how the performances of the PPIIP methods vary across the two different abstraction levels, i.e. exons versus residues, of PPI interfaces, we compare the methods’ performances on the EEI versus RRI prediction tasks (Section 1.5, available as supplementary data at *Bioinformatics Advances* online). For each PPIIP method, its EEI prediction performance is significantly (*q*-value <.05) better than its RRI prediction performance based on AU-PRC, MCC, precision, and F-score (Fig. 6, Fig. 12, available as supplementary data at *Bioinformatics Advances* online). In terms of recall, the RRI prediction performance of dMaSIF and GLINTER is significantly (*q*-value <.05) better than the EEI prediction performance for any FDR choice. The large differences in the precision of EEI versus RRI predictions could be related to the differences in their respective ratios of positive and negative interactions. That is, the RRI datasets are highly imbalanced with ∼350× more negative samples than positive samples, while the EEI datasets show much less data imbalance with only up to four times as many negative samples as positive samples. This high imbalance of the RRI datasets can make it challenging for the prediction models to correctly identify true interactions within a large number of negative instances.

### 3.6 Comparison of performance on experimental versus predicted protein 3D structures

To assess the applicability of our pre-trained pipelines on predicted protein structures, we evaluate the performance of one of them using protein structures from the AlphaFold Protein Structure Database (AFDB) (Fleming *et al.* 2025). We select the dMaSIF with $D_{\text{Con}}$ pre-trained pipeline as it gives the best performance in terms of AU-ROC based on PDB structures (Section 3.4). Here, we create an alternative $D_{\text{Con}}$ containing AFDB structures instead of the original PDB structures, as follows. For every PDB structure in $D_{\text{Con}}$, we select the corresponding AFDB protein structure if and only if the AFDB structure contains all residues of the PDB structure in the same sequence order. Then, for the resulting AFDB structures we keep the 3D coordinates of only those residues that are also present in the corresponding PDB structures. This strategy makes sure that we only use those parts of the predicted structures that are present in the PDB structures, for a fair evaluation of our pipeline on predicted structures. Then, we only keep a protein pair from $D_{\text{Con}}$ that has AFDB structures for both proteins as per the above criteria. This results in 349 out of the 504 protein pairs of $D_{\text{Con}}$ (Table 1), which we denote as $D_{\text{Con}}^{\text{small}}$. We then evaluate the dMaSIF with $D_{\text{Con}}$ pre-trained pipeline on the following three datasets: $D_{\text{Con}}$ with PDB structures, $D_{\text{Con}}^{\text{small}}$ with PDB structures ($D_{\text{Con}}^{\text{small}}-\text{PDB}$), and $D_{\text{Con}}^{\text{small}}$ with the AFDB structures ($D_{\text{Con}}^{\text{small}}-\text{AF}$). Recall that we have five pre-trained models for this pipeline and correspondingly five test sets (Section 2.4). Here, the protein pairs in the individual folds of $D_{\text{Con}}^{\text{small}}$ are exactly the same as those in the individual folds of $D_{\text{Con}}$ minus the protein pairs not found in AFDB. For each fold, we run the corresponding pre-trained model for the RRI and the EEI prediction tasks (using PPMax and PPDL), evaluating the performance based on AU-ROC.

**Figure 7:**
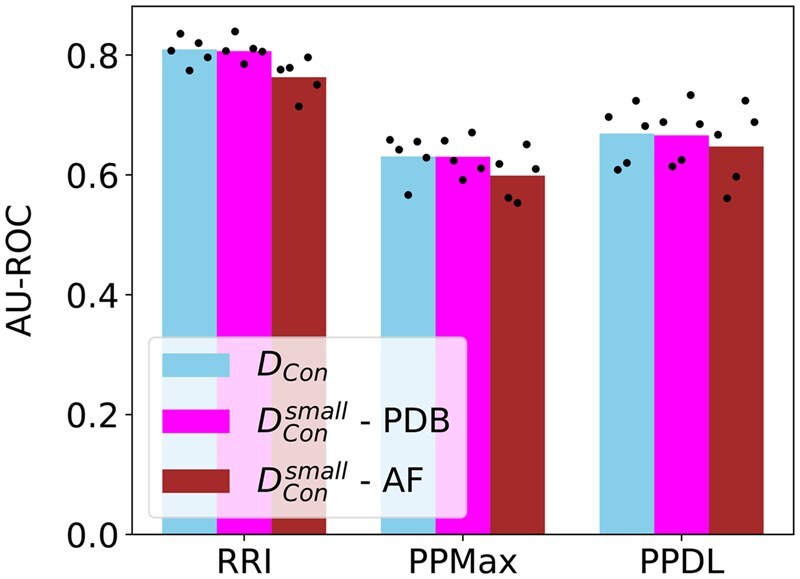
Performance evaluation of our dMaSIF with $D_{\text{Con}}$ pre-trained pipeline on the RRI prediction task (left) as well as the EEI prediction task using the PPMax (center), and PPDL (right), based on experimental and predicted protein structures. The height of the bars shows the mean AU-ROC, while the black points represent the performances for individual folds.

We find that the performances of our dMaSIF-based pre-trained models on predicted structures ($D_{\text{Con}}^{\text{small}}-\text{AF}$) are comparable (with marginal differences) to that on experimental structures ($D_{\text{Con}}^{\text{small}}-\text{PDB}$) (Fig. 7). Most importantly, for EEI prediction using PPDL our pipeline maintains high stability across PDB versus AFDB structures. These results highlight the potential of our pre-trained pipelines to utilize AFDB protein structures for predicting novel EEIs.

### 3.7 Prediction of novel EEIs in known PPIs

We use the best (in terms of precision with 1% FDR) EEI prediction model, i.e. ProteinMAE with PPDL post-processing trained on fold 5 of the $D_{\text{Engy}}$ dataset (Table 6, available as supplementary data at *Bioinformatics Advances* online), to predict novel EEIs in all 261 high-confidence human PPIs (but lacking the PPI interface information) that have been validated by multiple experimental methods (Section 1.1.6, available as supplementary data at *Bioinformatics Advances* online). Given a PPI, the model predicts an EEI score for each inter-protein exon pair between the corresponding two proteins. Among the 261 PPIs, there are 30 569 inter-protein exon pairs, and hence we obtain 30 569 predicted EEI scores (Table 7, available as supplementary data at *Bioinformatics Advances* online). To further assign confidence to these predicted scores in terms of FDR, we compare them to a background distribution constituting predicted scores of all non-interacting exon pairs from the set of interacting protein pairs on which the model was trained (i.e. the training set of fold 5 of the $D_{\text{Engy}}$ dataset). We find that for 436 of the 30 569 exon pairs, the EEI prediction scores are higher than the maximum prediction score of the background distribution (Fig. 13, available as supplementary data at *Bioinformatics Advances* online). The 436 predicted EEIs come from 111 PPIs (among 174 proteins), indicating that these novel EEIs are distributed across multiple PPIs.

To analyze the biological relevance of these predictions, we examine whether the predicted EEIs involve exons that are known to be alternatively spliced as per the information in UniProt. Of the 174 proteins, 32 proteins (covering 39 PPIs) have known isoforms where an entire exon is missing and that exon is part of a novel predicted EEI. From these 39 PPIs, we highlight two EEI examples. First, an EEI between proteins encoded by genes SNRNP200 [UniProt-ID (UID): O75643] and PRPF6 (UID: O94906) involving the exon with Ensembl ID ENSE00000963283 from SNRNP200 and exon ENSE00000856671 from PRPF6. SNRNP200 is part of the spliceosomal U4/U6.U5 tri-snRNP complex, and thus functionally relevant for AS. Skipping of ENSE00000963283 produces an alternative isoform (UID: O75643-2) of SNRNP200 (Bertram *et al.* 2017). Hence, the production of O75643-2 (instead of the canonical protein), could perturb the known PPI between SNRNP200 and PRPF6, and perhaps interfere with normal AS processes. Second, an EEI between proteins encoded by genes RAD51C (UID: O43502) and RAD51B (UID: O15315) involving exons ENSE00003481223 and ENSE00003579281, respectively. Both RAD51C and RAD51B are core components of the BCDX2 complex, essential for double-strand break repair (Somyajit *et al.* 2012). ENSE00003481223 is skipped in an isoform (O43502-2) of RAD51C (Chun *et al.* 2013, Rodrigue *et al.* 2013), indicating that the production of O43502-2 could perturb the known PPI between RAD51C and RAD51B, potentially also perturbing the BCDX2 complex.

### 3.8 Comparison of runtime

To evaluate the applicability of the trained EEI prediction models of the PPIIP methods for real-world usage on unseen data, we measure their runtime on a randomly chosen set of 10 protein pairs (Table 8, available as supplementary data at *Bioinformatics Advances* online). We limit ourselves to 10 protein pairs because we use the same set of proteins to evaluate the runtime of other, more time-consuming, PPIIP methods and protein complex structure prediction models which we do not include in our study further due to their long runtime (Table 8 and Section 1.2, available as supplementary data at *Bioinformatics Advances* online). We perform the analysis on a server running the operating system openSUSE Tumbleweed using one NVIDIA A40 GPU. We apply no restrictions regarding the number of threads.

Given the protein pairs, we first measure the pre-processing runtimes of the evaluated methods (Table 2). A much longer pre-processing runtime for GLINTER, AF-Multimer, and AF 3 comes partially from the multiple sequence alignment step, which is not required for dMaSIF, ProteinMAE, and PInet. Next, given the pre-processed protein pairs as input, we measure the prediction runtimes of the evaluated methods (Table 2). These are all in the same range for the PPIIP methods. So dMaSIF and ProteinMAE both have similar total (pre-processing + prediction) runtimes and are both much faster than PInet and GLINTER, and orders of magnitude faster than the AF models. For example, the total runtime of ProteinMAE is ∼96× and ∼10× lower than the total runtime for GLINTER and PInet, respectively, and ∼1700× and ∼350× lower than AF-Multimer and AF 3, respectively.

## 4 Conclusion

We extend four existing 3D structure-based PPIIP methods, i.e. dMaSIF, PInet, GLINTER, and ProteinMAE, originally designed for RRI predictions, to predict EEIs. Overall, the EEI prediction performances of the methods are significantly better than random. The output of our study can be used by the scientific community in two ways.

First, our computational pipelines can be used to predict EEIs between any two interacting proteins by (i) using one of our pre-trained models or (ii) training a model from scratch. While dMaSIF shows the highest AU-ROC, ProteinMAE with the PPDL post-processing EEI prediction pipeline shows the best precision-based performance with the lowest computational runtime. Hence, ProteinMAE should be the first choice to predict novel EEIs to reduce false positives. As an example, we use a pre-trained model of this pipeline to predict novel EEIs within a set of high-confidence human PPIs (Section 3.7). We train and test our EEI prediction pipelines using known 3D protein complexes from the PDB. As such, our pipelines are trained to distinguish between interacting versus non-interacting exon pairs across two proteins in a PPI. In general, our pipelines could be tested on protein pairs that do not interact, e.g. from the Negatome database (Blohm *et al.* 2014). Such evaluations represent an important consideration for future development of EEI prediction tools.

Second, our study provides insights into how to adapt existing PPIIP methods, designed for the RRI prediction task, for solving the EEI prediction task. Specifically, when using the RRI prediction scores of the existing PPIIP methods, we show that a CNN-based post-processing learns the patterns in the predicted RRI scores to correctly distinguish between interacting and non-interacting exon pairs. Other machine learning-based post-processing approaches might be used to more accurately capture such patterns and thus to further “fine-tune” the existing PPIIP methods for EEI predictions.

Such developments must also account for the data limitations faced by this study. A primary challenge is that there is no experimental ground truth specifically for EEIs. Because of this, we computationally derived a ground truth by mapping exons onto crystallized protein complexes. Another limitation is that PDB entries often contain only protein fragments. As a consequence, the training data may lack the complete structural context needed for accurate interface prediction, as models are trained on partial protein structures rather than full-length proteins. Further, PDB data are biased towards stable complexes, which could be reflected in our trained models. Despite these data constraints, the predicted EEIs could be integrated into existing computational frameworks, e.g. NEASE (Louadi *et al.* 2021) or DIGGER (Louadi *et al.* 2021), that explore the functional impact of the addition or removal of exons due to AS. Such methods overlay expressed exons from transcriptomics data onto an exon-level view of PPIs (i.e. EEIs) to predict perturbed biological processes. Thus, novel predicted EEIs could expand the current understanding of the functional impact of AS within AS-prone biological conditions.

## Supplementary Material

vbag032_Supplementary_Data

## Data Availability

All data used in this study were downloaded from a public database (The Protein Data Bank). Processed datasets and pre-trained models are available at https://doi.org/10.6084/m9.figshare.25416760. The source code is available at https://github.com/lieboldj/EEIpred.

## References

[vbag032-B1] Bender BJ , Gahbauer S, Luttens A et al A practical guide to large-scale docking. Nat Protoc 2021;16:4799–832. 34561691 10.1038/s41596-021-00597-z PMC8522653

[vbag032-B2] Berman HM , Westbrook J, Feng Z et al The protein data bank. Nucleic Acids Res 2000;28:235–42. 10592235 10.1093/nar/28.1.235 PMC102472

[vbag032-B3] Bertram K , Agafonov DE, Dybkov O et al Cryo-EM structure of a pre-catalytic human spliceosome primed for activation. Cell 2017;170:701–13.e11. 28781166 10.1016/j.cell.2017.07.011

[vbag032-B4] Bliven S , Lafita A, Parker A et al Automated evaluation of quaternary structures from protein crystals. PLoS Comput Biol 2018;14:e1006104. 29708963 10.1371/journal.pcbi.1006104 PMC5945228

[vbag032-B5] Blohm P , Frishman G, Smialowski P et al Negatome 2.0: a database of non-interacting proteins derived by literature mining, manual annotation and protein structure analysis. Nucleic Acids Res 2014;42:D396–400. 24214996 10.1093/nar/gkt1079 PMC3965096

[vbag032-B6] Bryant P , Pozzati G, Zhu W et al Predicting the structure of large protein complexes using AlphaFold and Monte Carlo tree search. Nat Commun 2022;13:6028. 36224222 10.1038/s41467-022-33729-4 PMC9556563

[vbag032-B7] Chun J , Buechelmaier ES, Powell SN et al Rad51 paralog complexes BCDX2 and CX3 act at different stages in the BRCA1-BRCA2-dependent homologous recombination pathway. Mol Cell Biol 2013;33:387–95. 23149936 10.1128/MCB.00465-12 PMC3554112

[vbag032-B8] Chung PJ , Choi MC, Miller HP et al Direct force measurements reveal that protein tau confers short-range attractions and isoform-dependent steric stabilization to microtubules. Proc Natl Acad Sci USA 2015;112:E6419–25. 10.1073/pnas.1513172112 PMC4664379 26542680

[vbag032-B9] Climente-González H , Porta-Pardo E, Godzik A et al The functional impact of alternative splicing in cancer. Cell Rep 2017;20:2215–26. 28854369 10.1016/j.celrep.2017.08.012

[vbag032-B10] Dai B , Bailey-Kellogg C. Protein interaction interface region prediction by geometric deep learning. Bioinformatics 2021;37:2580–8. 33693581 10.1093/bioinformatics/btab154 PMC8428585

[vbag032-B11] Dana JM , Gutmanas A, Tyagi N et al SIFTS: updated structure integration with function, taxonomy and sequences resource allows 40-fold increase in coverage of structure-based annotations for proteins. Nucleic Acids Res 2019;47:D482–9. 30445541 10.1093/nar/gky1114 PMC6324003

[vbag032-B12] Davis J , Goadrich M. The relationship between precision-recall and ROC curves. In: *Proceedings of the 23rd International Conference on Machine Learning-ICML ’06*, Pittsburgh, PA, USA, p.233–40. New York, NY, USA: ACM Press, 2006. 10.1145/1143844.1143874

[vbag032-B13] Durinck S , Moreau Y, Kasprzyk A et al BioMart and bioconductor: a powerful link between biological databases and microarray data analysis. Bioinformatics 2005;21:3439–40. 16082012 10.1093/bioinformatics/bti525

[vbag032-B14] Esmaielbeiki R , Krawczyk K, Knapp B et al Progress and challenges in predicting protein interfaces. Brief Bioinform 2015;17:117–31. 25971595 10.1093/bib/bbv027 PMC4719070

[vbag032-B15] Evans R, O’Neill M, Pritzel A, et al Protein complex prediction with AlphaFold-Multimer. bioRxiv; 2021. 10.1101/2021.10.04.463034

[vbag032-B16] Faisal FE , Newaz K, Chaney JL et al GRAFENE: graphlet-based alignment-free network approach integrates 3D structural and sequence (residue order) data to improve protein structural comparison. Sci Rep 2017;7:14890. 29097661 10.1038/s41598-017-14411-y PMC5668259

[vbag032-B17] Fleming J , Magana P, Nair S et al AlphaFold protein structure database and 3D-Beacons: new data and capabilities. J Mol Biol 2025;437:168967. 40133787 10.1016/j.jmb.2025.168967

[vbag032-B18] Ghadie MA , Lambourne L, Vidal M et al Domain-based prediction of the human isoform interactome provides insights into the functional impact of alternative splicing. PLoS Comput Biol 2017;13:e1005717. 28846689 10.1371/journal.pcbi.1005717 PMC5591010

[vbag032-B19] Gjerga E , Naarmann-de Vries IS, Dieterich C et al Characterizing alternative splicing effects on protein interaction networks with LINDA. Bioinformatics 2023;39:i458–64. 37387163 10.1093/bioinformatics/btad224 PMC10311343

[vbag032-B20] Goode BL , Chau M, Denis PE et al Structural and functional differences between 3-repeat and 4-repeat tau isoforms. J Biol Chem 2000;275:38182–9. 10984497 10.1074/jbc.M007489200

[vbag032-B21] Guo Z , Liu J, Skolnick J et al Prediction of inter-chain distance maps of protein complexes with 2D attention-based deep neural networks. Nat Commun 2022;13:6963. 36379943 10.1038/s41467-022-34600-2 PMC9666547

[vbag032-B22] Kagaya Y , Nakamura T, Verburgt J et al Structure modeling protocols for protein multimer and RNA in CASP16 with enhanced MSAs, model ranking, and deep learning. Proteins 2026;94:167–82. 40751131 10.1002/prot.70033 PMC12321240

[vbag032-B23] Krissinel E , Henrick K. Inference of macromolecular assemblies from crystalline state. J Mol Biol 2007;372:774–97. 17681537 10.1016/j.jmb.2007.05.022

[vbag032-B24] Levy SF , Leboeuf AC, Massie MR et al Three- and four-repeat tau regulate the dynamic instability of two distinct microtubule subpopulations in qualitatively different manners. J Biol Chem 2005;280:13520–8. 15671021 10.1074/jbc.M413490200

[vbag032-B25] Lin P , Yan Y, Huang S-Y et al DeepHomo2.0: improved protein–protein contact prediction of homodimers by transformer-enhanced deep learning. Brief Bioinform 2023a;24:bbac499. 36440949 10.1093/bib/bbac499

[vbag032-B26] Lin P , Tao H, Li H et al Protein–protein contact prediction by geometric triangle-aware protein language models. Nat Mach Intell 2023b;5:1275–84.

[vbag032-B27] Lin P , Yan Y, Tao H et al Deep transfer learning for inter-chain contact predictions of transmembrane protein complexes. Nat Commun 2023c;14:4935. 37582780 10.1038/s41467-023-40426-3 PMC10427616

[vbag032-B28] Louadi Z , Yuan K, Gress A et al DIGGER: exploring the functional role of alternative splicing in protein interactions. Nucleic Acids Res 2021;49:D309–18. 32976589 10.1093/nar/gkaa768 PMC7778957

[vbag032-B29] Louadi Z , Elkjaer ML, Klug M et al Functional enrichment of alternative splicing events with NEASE reveals insights into tissue identity and diseases. Genome Biol 2021;22:327. 34857024 10.1186/s13059-021-02538-1 PMC8638120

[vbag032-B30] Martin FJ , Amode MR, Aneja A et al Ensembl 2023. Nucleic Acids Res 2023;51:D933–41. 36318249 10.1093/nar/gkac958 PMC9825606

[vbag032-B31] Meng X-Y , Zhang H-X, Mezei M et al Molecular docking: a powerful approach for structure-based drug discovery. Curr Comput Aided Drug Des 2011;7:146–57. 21534921 10.2174/157340911795677602 PMC3151162

[vbag032-B32] Narykov O , Johnson NT, Korkin D et al Predicting protein interaction network perturbation by alternative splicing with semi-supervised learning. Cell Rep 2021;37:110045. 34818539 10.1016/j.celrep.2021.110045

[vbag032-B33] Newaz K , Ghalehnovi M, Rahnama A et al Network-based protein structural classification. R Soc Open Sci 2020;7:191461. 32742675 10.1098/rsos.191461 PMC7353965

[vbag032-B34] Newaz K , Piland J, Clark PL et al Multi‐layer sequential network analysis improves protein 3D structural classification. Proteins 2022;90:1721–31. 35441395 10.1002/prot.26349 PMC9356989

[vbag032-B35] Newaz K , Schaefers C, Weisel K et al Prognostic importance of splicing-triggered aberrations of protein complex interfaces in cancer. NAR Genom Bioinform 2024;6:lqae133. 39328266 10.1093/nargab/lqae133 PMC11426328

[vbag032-B36] Nikom D , Zheng S. Alternative splicing in neurodegenerative disease and the promise of RNA therapies. Nat Rev Neurosci 2023;24:457–73. 37336982 10.1038/s41583-023-00717-6

[vbag032-B37] Oughtred R , Rust J, Chang C et al The BioGRID database: a comprehensive biomedical resource of curated protein, genetic, and chemical interactions. Protein Sci 2021;30:187–200. 33070389 10.1002/pro.3978 PMC7737760

[vbag032-B38] Panda D , Samuel JC, Massie M et al Differential regulation of microtubule dynamics by three- and four-repeat tau: implications for the onset of neurodegenerative disease. Proc Natl Acad Sci USA 2003;100:9548–53. 12886013 10.1073/pnas.1633508100 PMC170955

[vbag032-B39] Rao J , Xie J, Yuan Q et al A variational expectation-maximization framework for balanced multi-scale learning of protein and drug interactions. Nat Commun 2024;15:4476. 38796523 10.1038/s41467-024-48801-4 PMC11530528

[vbag032-B40] Rodrigue A , Coulombe Y, Jacquet K et al The RAD51 paralogs ensure cellular protection against mitotic defects and aneuploidy. J Cell Sci 2013;126:348–59. 23108668 10.1242/jcs.114595

[vbag032-B41] Schmidhuber J. Deep learning in neural networks: an overview. Neural Netw 2015;61:85–117. 25462637 10.1016/j.neunet.2014.09.003

[vbag032-B42] Shoemaker BA , Panchenko AR. Deciphering protein–protein interactions. Part I. Experimental techniques and databases. PLoS Comput Biol 2007;3:e42. 17397251 10.1371/journal.pcbi.0030042 PMC1847991

[vbag032-B43] Si Y , Yan C. Protein language model-embedded geometric graphs power inter-protein contact prediction. Elife 2024;12:RP92184. 38564241 10.7554/eLife.92184 PMC10987090

[vbag032-B44] Somyajit K , Subramanya S, Nagaraju G et al Distinct roles of FANCO/RAD51C protein in DNA damage signaling and repair. J Biol Chem 2012;287:3366–80. 22167183 10.1074/jbc.M111.311241 PMC3270991

[vbag032-B45] Stark C , Breitkreutz B-J, Reguly T et al BioGRID: a general repository for interaction datasets. Nucleic Acids Res 2006;34:D535–9. 16381927 10.1093/nar/gkj109 PMC1347471

[vbag032-B46] Steinegger M , Söding J. MMseqs2 enables sensitive protein sequence searching for the analysis of massive data sets. Nat Biotechnol 2017;35:1026–8. 29035372 10.1038/nbt.3988

[vbag032-B47] Sun J , Frishman D. Improved sequence-based prediction of interaction sites in α-helical transmembrane proteins by deep learning. Comput Struct Biotechnol J 2021;19:1512–30. 33815689 10.1016/j.csbj.2021.03.005 PMC7985279

[vbag032-B48] Sverrisson F , Feydy J, Correia BE et al Fast end-to-end learning on protein surfaces. In: *2021 IEEE/CVF Conference on Computer Vision and Pattern Recognition (CVPR)*, Nashville, TN, USA, 2021, pp. 15267–15276. 10.1109/CVPR46437.2021.01502

[vbag032-B49] Tang M , Wu L, Yu X et al Prediction of protein–protein interaction sites based on stratified attentional mechanisms. Front Genet 2021;12:784863. 34880910 10.3389/fgene.2021.784863 PMC8647646

[vbag032-B50] Uniprot Consortium. UniProt: a worldwide hub of protein knowledge. Nucleic Acids Res 2019;47:D506–15. 30395287 10.1093/nar/gky1049 PMC6323992

[vbag032-B51] Velankar S , Dana JM, Jacobsen J et al SIFTS: structure integration with function, taxonomy and sequences resource. Nucleic Acids Res 2013;41:D483–9. 23203869 10.1093/nar/gks1258 PMC3531078

[vbag032-B52] Wright CJ , Smith CWJ, Jiggins CD et al Alternative splicing as a source of phenotypic diversity. Nat Rev Genet 2022;23:697–710. 35821097 10.1038/s41576-022-00514-4

[vbag032-B53] Xie Z , Xu J. Deep graph learning of inter-protein contacts. Bioinformatics 2022;38:947–53. 34755837 10.1093/bioinformatics/btab761 PMC8796373

[vbag032-B54] Xue LC , Dobbs D, Bonvin AMJJ et al Computational prediction of protein interfaces: a review of data driven methods. FEBS Lett 2015;589:3516–26. 26460190 10.1016/j.febslet.2015.10.003 PMC4655202

[vbag032-B55] Yang X , Coulombe-Huntington J, Kang S et al Widespread expansion of protein interaction capabilities by alternative splicing. Cell 2016;164:805–17. 26871637 10.1016/j.cell.2016.01.029 PMC4882190

[vbag032-B56] Yuan M , Shen A, Fu K et al ProteinMAE: masked autoencoder for protein surface self-supervised learning. Bioinformatics 2023;39:btad724. 38019955 10.1093/bioinformatics/btad724 PMC10713117

[vbag032-B57] Yuan Q , Chen J, Zhao H et al Structure-aware protein–protein interaction site prediction using deep graph convolutional network. Bioinformatics 2021;38:125–32. 34498061 10.1093/bioinformatics/btab643

[vbag032-B58] Zeng M , Zhang F, Wu F-X et al Protein–protein interaction site prediction through combining local and global features with deep neural networks. Bioinformatics 2020;36:1114–20. 31593229 10.1093/bioinformatics/btz699

[vbag032-B59] Zhang Y , Qian J, Gu C et al Alternative splicing and cancer: a systematic review. Signal Transduct Target Ther 2021;6:78. 33623018 10.1038/s41392-021-00486-7 PMC7902610

