# Supplementary material for: The power and limits of predicting inter-protein exon-exon interactions using protein 3D structures
Source: Bioinform Adv. 2026 Jan 27;6(1):vbag032. doi: 10.1093/bioadv/vbag032 (PMC12974993; doi:10.1093/bioadv/vbag032)
Supplement: vbag032_Supplementary_Data [file vbag032_supplementary_data.zip › EEIP_Supp_Materials.pdf]

Supplementary materials: The power and limits of predicting inter-protein exon-exon interactions using protein 3D structures

Jeanine Liebold[1,2], Aylin Del Moral-Morales[1,3], Karen Manalastas-Cantos[1,4,5], Olga Tsoy[1,6], Stefan Kurtz[2], Jan Baumbach[1,7,*], Khalique Newaz[1,5,*]

[1]Institute for Computational Systems Biomedicine, Universität Hamburg, 22761 Hamburg, Germany,
[2]Faculty of Mathematics, Informatics and Natural Sciences, Center for Bioinformatics, Universität Hamburg, 22761 Hamburg, Germany,
[3]Departamento de Ciencias Naturales, Universidad Autónoma Metropolitana-Cuajimalpa, Mexico City, Mexico,
[4]Leibniz-Institut für Virologie (LIV), Centre for Structural Systems Biology (CSSB), 22607 Hamburg, Germany,
[5]Center for Data and Computing in Natural Sciences, Universität Hamburg, 22761 Hamburg, Germany,
[6]Department of Computer Science, Bioinformatics, Vrije Universiteit Amsterdam, Amsterdam, The Netherlands,
[7]Department of Mathematics and Computer Science, University of Southern Denmark, 5230 Odense, Denmark

* Corresponding authors

1 Supplementary Sections

1.1 Dataset details

1.1.1 Collection and preprocessing of PDB data

We consider all of those 5,977 PDB entries (downloaded on January 2022) that contain at least one pair of reviewed human proteins from UniProt, that are co-resolved (i.e., form a potential heterodimer), and that provide sufficient 3D structural resolution (less than or equal to 3 Å) information. Because of the redundancy in the PDB data, one PDB entry can contain multiple protein pairs or one protein pair can occur in multiple PDB entries. Consequently, the 5,977 PDB entries capture 13,967 unique protein pairs with possible multiple occurrences for every protein pair. To choose one representative PDB entry for a given protein pair, we do the following. First, we select all PDB entries with the highest number of the resolved amino acids of the two proteins. Second, if multiple PDB entries remain after the first step, then we choose the one with the highest 3D resolution. The above steps result in 2,349 unique PDB entries with information about 13,967 unique co-resolved protein pairs among 2,882 unique proteins. We download the Crystallographic Information Files (CIFs) containing 3D coordinates of amino acid atoms of proteins of the corresponding PDB entries to use in the subsequent analyses.

1.1.2 Mapping exon information onto protein sequences

For each of the 2,882 proteins, we download the corresponding amino acid sequences from the UniProt database (UniProt Consortium, 2019). To map exon information onto each of the 2,882 UniProt protein sequences using the Ensembl database (Martin *et al.*, 2023), we do the following: First, we use the BiomaRt package (Durinck *et al.*, 2005) in R to map UniProt IDs to the Ensembl transcripts. Because for many genes the Ensembl

database has information for more than one transcript, there is no one-to-one mapping between our UniProt protein sequences and the corresponding Ensembl transcripts. To select a unique transcript corresponding to each protein in our set of protein sequences, we select the Ensembl transcript of the corresponding gene that aligns entirely (i.e., with no gaps and no mismatches) with the given protein sequence based on Needleman-Wunsch pairwise global sequence alignment, which we perform using the Biostrings R package (H. Pagès, P. Aboyoun, R. Gentleman, and S. DebRoy, 2017). If we do not find any such transcript, then we exclude the corresponding UniProt sequence from our study. Out of the 13,967 co-resolved protein pairs, we keep only those pairs (p_1, p_2) where the sequences of each of the proteins p_1 and p_2 have a unique Ensembl transcript as per the criteria defined above. This results in 2,087 unique PDB entries that contain information about the co-crystallization of 13,235 (out of the 13,967) protein pairs, containing 2,660 unique proteins. After identifying a unique Ensembl transcript ID for each of the final set of 2,660 UniProt protein sequences, we use the Ensembl GTF file ("Homo_sapiens.GRCh38.105.gtf.gz" downloaded on January 2022) to map the exon information corresponding to the transcript IDs on to the corresponding protein sequences. Note that because Ensembl provides exon information in terms of DNA coordinates, a codon may be split between two exons. If this happens, we assign the codon the exon ID to which two of its three nucleotides belong.

1.1.3 Mapping PDB information onto UniProt sequences

For each of the 2,660 proteins, we map their amino acid sequence positions onto their 3D resolved structures from the corresponding PDB entries using the Structure integration with function, taxonomy, and sequence (SIFTS) database (Dana *et al.*, 2019; Velankar *et al.*, 2013). Given a PDB entry, the SIFTS database provides the corresponding UniProt protein ID and the one-to-one mapping between the amino acid sequence positions of the UniProt protein ID and the amino acid sequence positions of the resolved 3D protein structure in the PDB. Among the 2,660 proteins, we only keep those proteins for which there is data in the SIFTS database. This results in 2,649 unique proteins across 2,064 unPDB entries with information about the co-crystallization of 13,190 unique protein pairs.

1.1.4 Exon-exon interaction definition

As outlined above, our data contains 2,064 PDB entries with 13,190 unique co-resolved protein pairs among 2,649 proteins. Given the set of data, as outlined in Section 2 of the main paper, for each pair of exons e1 from p1 and e2 from p2, we define two exons to form an exon-exon interaction (EEI) using three approaches, i.e., contact-based, energy-based, and evolution-based. Here, we extend the explanation with relevant details.

Contact-based. We define an EEI between two exons e_1 and e_2 if and only if at least one residue of e_1 interacts with at least one residue of e_2. We define an interaction between a pair of residues if and only if the 3D Euclidean distance between any of the heavy atoms (i.e., Carbon, Nitrogen, Oxygen, and Sulphur) of r_1 is within 6 Å to any of the heavy atoms of r_2. Among 2,064 PDB entries, we only consider those 1,543 entries that (1) contain at least one interacting exon pair and (2) could be preprocessed by each of the four PPIIP methods (Section 2.2). For training, among 13,190 co-resolved protein pairs, 2,231 protein pairs remain that contain 10,793 interacting exon pairs. In total 3,493 PDB chains are involved in

the 2,231 protein pairs. We take all remaining inter-protein exon pairs within each of the 2,231 protein pairs as non-interacting, resulting in 38,060 non-interacting exon pairs. Among the 10,793 EEIs, we obtain 165,744 interacting and 62,216,726 non-interacting residue pairs. For testing, among 13,190 co-resolved protein pairs, 504 protein pairs remain that contain 2,658 interacting exon pairs. In total 906 PDB chains are involved in the 504 protein pairs. We take all remaining inter-protein exon pairs within each of the 504 protein pairs as non-interacting, resulting in 9,552 non-interacting exon pairs. Among the 2,658 EEIs, we obtain 40,196 interacting and 15,301,839 non-interacting residue pairs.

Energy-based. We use Protein Interfaces, Surfaces, and Assemblies (PISA) (Krissinel and Henrick, 2007) to identify biologically relevant interfaces of co-resolved proteins. PISA first computes a solvation free energy gain of an interface, which quantifies the thermodynamic changes that occur due to the interface formation. Then it computes the probability of observing the same solvation free energy gain by chance when a random set of atoms (with the same area as that of the interface) is picked from the non-interfacing surfaces of the two proteins. PISA labels an interface as biologically relevant if the corresponding probability is less than 0.5. We define an EEI between e_1 and e_2 if and only if they overlap with biologically relevant interfaces. Among 2,064 PDB entries, we only consider those 1,321 entries that (1) contain at least one co-resolved protein pair with at least one interacting exon pair and (2) could be preprocessed by each of the four PPIIP methods. For training, among 13,190 co-resolved protein pairs, 1,580 protein pairs remain that contain 15,049 interacting exon pairs. In total 2,977 PDB chains are involved in the 1,580 protein pairs. We take all remaining inter-protein exon pairs within each of the 1,580 protein pairs as non-interacting, resulting in 28,227 non-interacting exon pairs. Among the 15,049 EEIs, we obtain 135,241 interacting and 54,918,541 non-interacting residue pairs. For testing, among 13,190 co-resolved protein pairs, 343 protein pairs remain that contain 3,112 interacting exon pairs. In total 662 PDB chains are involved in the 343 protein pairs. We take all remaining inter-protein exon pairs within each of the 343 protein pairs as non-interacting, resulting in 4,700 non-interacting exon pairs. Among the 3,112 EEIs, we obtain 30,250 interacting and 10,423,823 non-interacting residue pairs.

Evolution-based. We use the Evolutionary Protein-Protein Interface Classifier (EPPIC) (Bliven *et al.*, 2018) to identify biologically relevant interfaces of co-resolved proteins. EPPIC characterizes an interface as biologically relevant if the interface surface area is more than 2,200 $Å^2$, while it characterizes an interface to be an artifact of crystallization if the interface surface area is less than 400 $Å^2$. For interface surface areas between 400 $Å^2$ and 2,200 $Å^2$, EPPIC uses information about the evolutionary conservation of the corresponding protein sequences to characterize whether the interface is biologically relevant or not. For our study, we run EPPIC using default parameters. We define an EEI between e_1 and e_2 if and only if they overlap with biologically relevant interfaces. Among 2,064 PDB entries, we only consider those 898 entries that (1) contain at least one co-resolved protein pair with at least one interacting exon pair and (2) could be preprocessed by each of the four PPIIP methods (Section 2.2). For training, among 13,190 co-resolved protein pairs, 970 protein pairs remain that contain 11,697 interacting exon pairs. In total 2,003 PDB chains are involved in the 970 protein pairs. We take all remaining inter-protein exon pairs within each

of the 970 protein pairs as non-interacting, resulting in 20,710 non-interacting exon pairs. Among the 11,697 EEIs, we obtain 114,778 interacting and 42,317,056 non-interacting residue pairs. For testing, among 13,190 co-resolved protein pairs, 231 protein pairs remain that contain 2,255 interacting exon pairs. In total 454 PDB chains are involved in the 231 protein pairs. We take all remaining inter-protein exon pairs within each of the 231 protein pairs as non-interacting, resulting in 3,852 non-interacting exon pairs. Among the 2,255 EEIs, we obtain 25,309 interacting and 8,178,273 non-interacting residue pairs.

1.1.5 MMSeqs2-based PDB chain clustering with inter-cluster pairwise sequence identity of <30%

To avoid data leakage between training and testing of the PPIIP models due to high sequence identity between proteins, we do the following. Given all PDB chains in a dataset (either contact-based, energy-based, or evolution-based), we use the many-against-many sequence searching tool MMSeqs2 (Steinegger and Söding, 2017) to obtain sequence non-redundant clusters of PDB chains (Bernett *et al.*, 2024). This results in 892 clusters for the contact-based (named D_{Con}), 728 clusters for the energy-based (named D_{Engy}), and 420 clusters (named D_{Evol}) for the evolution-based datasets. MMSeqs2 is a computationally efficient sequence identity quantification tool that has been shown to acquire similar accuracy as traditional alignment methods such as BLAST (Altschul *et al.*, 1990). We run MMSeqs2 using the command *mmseqs easy-cluster --min-seq-id 0.3 -c 0.8 --cov-mode 0*, which ensures that any pairwise sequence identity between PDB chains within a cluster is at least 30% with at least 80% of the residues aligned, while any pairwise sequence identity between PDB chains across clusters is below 30%. Note that the 30% sequence identity threshold is commonly used to ensure that protein sequences across different clusters are potentially non-homologous (Rost, 1999).

1.1.6 Independent dataset for generating novel EEI predictions

We consider all of those human PPIs from BioGRID (Version 4-4-244) (Oughtred *et al.*, 2021) that are experimentally verified by at least the following four experimental systems: two-hybrid, affinity capture-western, reconstituted complex, and affinity capture-mass spectrometry. From this set of PPIs, we select a PPI in which each of its participating proteins (1) is from the 20,378 reviewed human proteins from UniProt (UniProt Consortium, 2019) (downloaded on January 2022), (2) is present in the AlphaFold Protein Structure Database (Varadi *et al.*, 2022), and (3) has exon annotations in Ensembl (Martin *et al.*, 2023). Furthermore, we exclude a PPI if any of its proteins is present in any of our three datasets used for performance evaluation of the PPIIP methods (Section 2.1). This results in 261 PPIs with 30,569 inter-protein exon-exon pairs.

1.2 PPI interface prediction methods

We consider 10 PPIIP methods (Supplementary Table S1) of which we could only run four, i.e., dMaSIF, GLINTER, PInet, and ProteinMAE, which have (1) their corresponding code publicly available and (2) reasonable prediction runtime per protein pair (< 10 minutes). To evaluate the runtime, we randomly choose 10 protein pairs. Next, for each method, we use a provided pre-trained model, load the given weights and perform an initial runtime analysis of the inference including the pre-processing for the 10 protein pairs. We notice

that some methods have a very time-consuming pre-processing procedure with > 3 hours for 10 proteins (Supplementary Table S8). Below, for each of the considered four PPIIP methods, we explain how we use them in our study to predict RRIs. We run all methods with their recommended parameter values to preprocess the input files and train the corresponding models.

1.2.1 Differentiable molecular surface interaction fingerprinting (dMaSIF)

dMaSIF (Sverrisson *et al.*, 2021) takes two proteins p_1 and p_2 as input where, for each protein, the type and the 3D coordinates of each atom are represented in the same coordinate space in the form of a ".pdb" file from the PDB database (Berman *et al.*, 2000) (or henceforth simply referred to as a PDB file). A PDB file generally does not contain information about the 3D coordinates of hydrogen atoms, which is required by dMaSIF. Hence, to preprocess the PDB files to add hydrogen atoms, we run the program Reduce (Word *et al.*, 1999) (using the command "*reduce -build -Quiet*"). Given the preprocessed input PDB files of two proteins, dMaSIF computes the point cloud representation of each of the two proteins, where it initializes each point in a point cloud by a feature vector of size 16. This vector consists of ten geometric and six physicochemical features summarized over 16 atoms in the corresponding protein nearest to the given point. dMaSIF considers two points x_1 and x_2 (where x_1 comes from protein p_1 and x_2 comes from protein p_2) to interact if their 3D Euclidean distance is < 1 Å. Finally, given the point clouds of the two proteins with initial feature vectors for each point and the knowledge about which two points of the two point clouds interact, dMaSIF trains a geometric deep learning model. Once the model is trained, given two test proteins q_1 and q_2 in the form of preprocessed PDB files (not necessarily represented in the same coordinate space) as input to the trained model, dMaSIF computes the point clouds of the two proteins and predicts a feature vector for each point of the two point clouds. dMaSIF takes the inner product of the feature vectors of each pair of points y_1 and y_2 (where y_1 comes from q_1 and y_2 comes from q_2) and recognizes the product values as the interaction scores of the corresponding point pairs, where a higher score means a higher chance of the two points to interact. Similarly, dMaSIF assigns interaction scores to each pair of atoms a_1 and a_2 between the proteins q_1 and q_2, where a higher score means a higher chance for the two atoms to interact. For more details please refer to the dMaSIF paper (Sverrisson *et al.*, 2021). We use dMaSIF to predict RRI scores for each pair of residues r_1 and r_2 (where r_1 comes from q_1 and r_2 comes from q_2) as the maximum of all interaction scores of all atom pairs a_i and a_j, where atom a_i belongs to r_1 and atom a_j belongs to r_2. To predict interaction scores between exons, we use two approaches as outlined in Supplementary Section 1.3.

1.2.2 Protein Interface Network (PInet)

PInet (Dai and Bailey-Kellogg, 2021) takes two proteins p_1 and p_2 as input where, for each protein, the type and the 3D coordinates of each atom are represented in the same coordinate space in the form of a PDB file. Before the model training process, PInet requires preprocessing of PDB files to add missing atoms or remove solvent molecules and to create point cloud representations of the two proteins. We follow the recommendations of PInet to preprocess the PDB files in our data. That is, we use the program PDB2PQR 3.5.2 (Dolinsky *et al.*, 2007) (using the command *"pdb2pqr30 x.pdb x.pqr --whitespace*

--ff=AMBER --apbs-input x.in", where *x* is any PDB ID) and APBS 1.5 (Baker *et al.*, 2001) (using the command "*apbs x.in*") to reconstruct missing atoms or remove solvent molecules. Additionally, we use the program PyMOL 2.5.4 (DeLano and Others, 2002) (using the command "*pymol -c -d "load [pdb file]; set surface_quality, 0; show_as surface, all; set_view 1,0,0,0,1,0,0,0,1,0,0,0,0,0,0,300,1; save [pdb file id].wrl; delete all"*") to create point cloud representations of the two proteins. PInet initializes each point in a point cloud by a feature vector of size five consisting of three geometric and two physicochemical features summarized over residues within a predefined neighborhood (within the corresponding protein) of the point. PInet considers two points x_1 and x_2 (where x_1 comes from protein p_1 and x_2 comes from protein p_2) to interact if their 3D Euclidean distance is < 2 Å. Given the point clouds of the two proteins with initial feature vectors for each point and the knowledge about which two points of the two point clouds interact, PInet trains a geometric deep learning model. Once the model is trained, given two test proteins q_1 and q_2 in the form of preprocessed PDB files or point clouds (not necessarily represented in the same coordinate space) as input to the trained model, for each point in each of the two point clouds, PInet predicts a score indicating the likelihood of the point to be part of the PPI interface. Similar to a score for a point, we assign a score for every residue in the two proteins indicating the likelihood of a residue to participate in the PPI interface by taking the average of the scores of the points nearest to the residue as proposed by PInet. For more details please refer to the PInet paper (Dai and Bailey-Kellogg, 2021). We use PInet to predict RRI scores for each pair of residues r_1 and r_2 (where r_1 comes from q_1 and r_2 comes from q_2) as the product of the individual probability scores of r_1 and r_2. To predict interaction scores between exons, we use two approaches as outlined in Supplementary Section 1.3.

1.2.3 Graph Learning of INTER-protein contacts (GLINTER)

GLINTER (Xie and Xu, 2022) takes two proteins p_1 and p_2 as input where, for each protein, the type and the 3D coordinates of each atom are represented in the same coordinate space in the form of a PDB file. Before the model training process, GLINTER requires the preprocessing of PDB files to create graph representations of each of the two proteins on three different levels, i.e., residue, atom, and surface. We follow the recommendations of GLINTER to create such graph representations. We use the suggested pretrained protein language model to get a contact map for a protein pair. To do this, we first run multiple sequence alignment (MSA) of each protein in our data using hhblits-bin with the uniclust database A3M_SpecBloc 2016/09 database (Mirdita *et al.*, 2016). Then, we use the concatenated joint MSA as input for the pre-trained ESM model ESM-MSA (Rao *et al.*, 18--24 Jul 2021) to create a symmetrized attention/contact map. For the graphs, we use the programs Reduce (Word *et al.*, 1999) (using the command "*reduce -Trim -HIS*"), MSMS version 2.6.1 (Sanner *et al.*, 1996) (using the command "*msms -density 3.0 -hdensity 3.0 -probe 1.5 -if $xyzrn -of $file_base -af $file_base*"), and the python package Trimesh version 3.21.5 (Dawson-Haggerty) to create surface graphs and the python script provided by GLINTER to create the residue and atom graphs. Given graph representations of the two input proteins, GLINTER initializes each node of each graph based on geometric, evolutionary, and physicochemical features. GLINTER considers two residues r_1 and r_2 of the input proteins p_1 and p_2 (where r_1 comes from protein p_1 and r_2 comes from protein p_2) to interact if their 3D Euclidean distance is < 8 Å. Finally, given all of the graphs of the two

proteins with initial feature vectors for each node, the output of the MSA transformer (a contact map), and the knowledge about which two residues of the two proteins interact, GLINTER trains a model. Given two test proteins q_1 and q_2 in the form of preprocessed PDB files (i.e. graphs and MSAs) as input to the trained model, for each residue pair r_1 (from protein q_1) and r_2 (from protein q_2), GLINTER predicts a score indicating the likelihood of the two residues to interact (for more details please refer to the actual paper (Xie and Xu, 2022)), which is what we use as an RRI score. The higher the RRI score, the higher the likelihood that the corresponding residue pairs interact. To predict interaction scores between exons, we use two approaches as outlined in Supplementary Section 1.3.

1.2.4 Protein Masked AutoEncoder (ProteinMAE)

ProteinMAE (Yuan *et al.*, 2023) takes two proteins p_1 and p_2 as input where, for each protein, the type and the 3D coordinates of each atom are represented in the same coordinate space in the form of a PDB file. Like dMaSIF (see Supplementary Section 1.2.1), ProteinMAE represents each protein as a point cloud where each point in a point cloud is described by a feature vector of size 16 consisting of ten geometric and six chemical features summarized over 16 atoms in the corresponding protein nearest to the given point. ProteinMAE considers two points x_1 and x_2 (where x_1 comes from protein p_1 and x_2 comes from protein p_2) to interact if their 3D Euclidean distance is < 1 Å. Similar to previous steps, the neural network architecture and the training procedure is adapted from dMaSIF, except for the complementary last layers, where ProteinMAE uses the same weights for both input proteins. The main difference between ProteinMAE and dMaSIF is that ProteinMAE specifically designs a self-supervised framework for protein surface representation, which enables the use of unlabeled data for pretraining. To train a ProteinMAE model for the RRI prediction task, as recommended by the original ProteinMAE study, we initialize the weights for the MAE-Encoder using the weights from the pre-trained model and initialize the other layers using the default in PyTorch (Paszke *et al.*, 2019). Given two test proteins q_1 *and* q_2, for each pair of residues r_1 and r_2 (where r_1 comes from q_1 and r_2 comes from q_2), ProteinMAE predicts RRI scores as the maximum of interaction scores of all atom pairs a_i and a_j, where a_i belongs to r_1 and a_j belongs to r_2. The higher the RRI score, the higher the likelihood that the corresponding residue pairs interact. For more details please refer to ProteinMAE's code base on GitHub as the corresponding paper (Yuan *et al.*, 2023) lacks sufficient details regarding RRI prediction. To predict interaction scores between exons, we use two approaches as outlined in Supplementary Section 1.3.

1.3 Post-processing via deep learning (PPDL)

As outlined in Section 2.3 of the main paper, we use a post-processing approach based on a convolutional neural network (PPDL) where, given a set of interacting and non-interacting exon pairs, it first trains a model to learn distinguishing patterns of the predicted RRI scores for interacting vs. non-interacting exons. Then, given two test exon pairs, it predicts the corresponding EEI score. Here, we outline further details about the approach.

To create inputs for PPDL, given a PPIIP method, for each pair of exons e_1 and e_2 with m and n residues respectively, we use the PPIIP method to obtain an $m \times n$ matrix $M(e_1,e_2)$. An entry $M(e_1,e_2)[i,j]$ is the predicted RRI score of the ith residue in e_1 and the jth residue in e_2. We focus on only those pairs (e_1,e_2) such that the number of residues in e_1 and the number of residues in e_2 are not larger than 100. For each of our datasets (Section 2.1), this selection criterion is satisfied by more than 95% of the interacting exon pairs. See Supplementary Table S3 for detailed numbers for interacting and non-interacting exon pairs. The input to our PPDL approach is a 100×100-matrix $M(e_1,e_2)$ corresponding to each exon pair (e_1,e_2). For exons shorter than 100, we use zero padding. The output of PPDL is a continuous score ranging from 0 to 1 where a higher score means a higher likelihood of interaction. Our PPDL architecture consists of two convolutional layers, two max-pooling layers, two non-linear activations for feature extraction, and two linear layers for the final classification (Supplementary Figure S1).

1.4 Model training, validation, and test set creation

For each of D_{Con}, D_{Engy}, and D_{Evol}, we first split the corresponding clusters into five non-overlapping subsets (or folds) using a round-robin forward-backward assignment. Formally, let $C = [c_0, c_1, \ldots, c_{n-1}]$ be the list of n clusters sorted in ascending order of size ($|c_0| \leq |c_1| \leq \ldots \leq |c_{n-1}|$). We define a function *subset_idx(i)* that determines to which of the five subsets the i-th cluster (c_i) is assigned, as follows:

$$\text{subset\_idx}(i) = \begin{cases} i \bmod 5 & \text{if } \lfloor \frac{i}{5} \rfloor \bmod 2 = 0 \\ 5 - 1 - (i \bmod 5) & \text{if } \lfloor \frac{i}{5} \rfloor \bmod 2 = 1 \end{cases}$$

We follow the above heuristic to keep the number of PDB chains across the five subsets as similar as possible. We use each subset as an independent test set (henceforth, referred to simply as “test set” for brevity) and the remaining four subsets for validation (one subset) and training (three subsets). Thus, in total, we use five combinations of training, validation, and test sets. Within a training, validation, or test set, we consider all co-resolved protein pairs. See Supplementary Table S3 for details regarding the numbers of clusters, PDB chains, protein pairs, EEIs, non-EEIs, as well as RRIs and non-RRIs, for each of the five combinations (or folds).

For RRI predictions, given the three datasets, the split of each dataset into five combinations of training, validation, and test sets, and four PPIIP methods, we train and test 3×5×4=60 RRI prediction models.

For EEI predictions, given a PPIIP method, we first use a model trained for RRI predictions to obtain the RRI scores for each protein pair in the corresponding test data. We do this for each of the five trained models corresponding to each of the five test sets, which gives RRI scores for each protein pair contained in any of the test sets. Then we use the predicted RRI scores to obtain the EEI scores using two post-processing approaches (Section 2.3). Because we use three datasets, a split of each dataset into five combinations of training, validation, and test sets, four PPIIP methods, and two post-processing approaches, we train and test 3×5×4×2=120 EEI prediction models.

1.5 Evaluation metrics

1.5.1 Performance metrics and decision thresholds

We evaluate each of the trained models using six performance measures: AU-ROC, AU-PRC, MCC, precision, recall, and F-score. While AU-ROC and AU-PRC can be computed using all prediction scores of the test data, the computation of MCC, precision, recall, and F-score requires a predefined score threshold (i.e., "decision threshold"), such that residue (or exon) pairs are considered to be interacting if and only if they achieve a score larger than the decision threshold. We define such a decision threshold as follows. For each fold, we take the predictions of all non-interacting residue (or exon) pairs from the training set as our background distribution of non-interacting residue (or exon) pair scores (Supplementary Figure S2). Then, we define a decision threshold as the score that accepts t% of non-interacting residue (or exon) pairs (i.e., false positives) from this background with t (i.e., the FDR) varying from 1 to 5 in increments of 1.

1.5.2 Statistical significance of performance differences between PPIIP methods in a prediction task

We compare the performance of any two PPIIP methods across all combinations of the three datasets (i.e., either D_{Con}, D_{Engy}, or D_{Evol}) and the five test sets (15 performance values in total), using the paired Wilcoxon signed-rank test. For each of the four threshold-dependent performance measures (either MCC, precision, recall, or F-score), we evaluate the performance separately at each of the five FDR choices (1%, 2%, 3%, 4%, and 5%), resulting in 4 × 5 = 20 performance tests in total for a pair of PPIIP methods. In addition, we evaluate the performance using two threshold-independent performance measures, AU-ROC and AU-PRC. Thus, for a pair of PPIIP methods, in total, we do 20 + 2 = 22 performance tests, resulting in 22 p-values. Given the six PPIIP method pairs derived from the four PPIIP methods, we get 22 x 6 = 132 p-values, which we correct using the Benjamini-Hochberg procedure to obtain the corresponding q-values.

1.5.3 Statistical significance of performance differences of a PPIIP method across prediction tasks or post-processing approaches

For each PPIIP method, we compare its performance across two prediction tasks. Similar to Supplementary Section 1.5.2, we evaluate the performance across all combinations of the three datasets (i.e., either D_{Con}, D_{Engy}, or D_{Evol}) and the five test sets (15 performance values in total), using the paired Wilcoxon signed-rank test. Also similar to Supplementary Section 1.5.2, we consider both threshold-dependent and threshold-independent performance measures, resulting in 22 performance tests. Given the four PPIIP methods and 22 performance tests, we get 4 x 22 = 88 p-values which we correct using the Benjamini-Hochberg procedure to obtain the corresponding q-values.

1.6 Structural characteristics of proteins

We annotate each residue of a protein based on secondary structural and intrinsically disordered region (IDR) information. For the secondary structural information, given a 3D protein structure, we extract its secondary structural characteristics using the dictionary of protein secondary structure (DSSP) (Kabsch and Sander, 1983). DSSP annotates each residue of a protein as one of the eight categories: α-helix, 3-helix, 5-helix, residue in

isolated β-bridge, extended strand participating in β ladder, hydrogen-bonded turn, bend, and undetermined output. Given a protein and the corresponding DSSP categories, we label each residue of the protein as either α-helix (if the corresponding DSSP annotation is either α-helix, 3-helix, or 5-helix), β-sheet (if the corresponding DSSP annotation is either residue in isolated β-bridge or extended strand participating in β ladder), or coil-turn (if the corresponding DSSP annotation is either hydrogen-bonded turn, bend, or undetermined output). For the IDR information, given a protein, we identify residues belonging to an IDR using the StrIDR database (Majila and Viswanath, 2024). StrIDR collects IDR information from three established IDR databases, i.e., DisProt (Aspromonte *et al.*, 2024), IDEAL (Fukuchi *et al.*, 2012), and MobiDB (Piovesan *et al.*, 2023), and maps the corresponding UniProt sequences to the PDB structures using SIFTS.

Given the secondary structural annotations of proteins, for each interacting and non-interacting exon pair, we compute the fraction of residues in the two exons combined that belong to a secondary structural label, i.e., α-helix, β-sheet, and coil-turn. Similarly, given the residue-level IDR annotations of proteins, for each interacting and non-interacting exon pair, we compute the fraction of residues in the two exons combined that are in an IDR region. Given a dataset, we evaluate the differences between interacting and non-interacting exon pairs based on each of the three secondary structural labels and the IDR content using the Mann-Whitney U (McKnight and Najab, 2010) and Kolmogorov-Smirnov (Massey, 1951) tests to obtain the corresponding p-values. We choose these tests to assess differences in the median (Mann-Whitney U) and overall distributional characteristics (Kolmogorov-Smirnov). For each test (i.e., Mann-Whitney U or Kolmogorov-Smirnov), we correct the p-values across all datasets, secondary structural labels, and IDR content together (3 x 4 = 12 p-values) using the Benjamini-Hochberg procedure to obtain the corresponding q-values.

1.7 Additional results

1.7.1 Dataset overlap and structural characterization of exon pairs

For each of D_{Con}, D_{Engy}, and D_{Evol}, we first obtain the corresponding set of protein pairs by taking the union of all protein pairs across all of the five test sets of PDB chain clusters (Section 2.4). Given the three sets of protein pairs, we (1) calculate their overlap in terms of protein and exon pairs to quantify the amount of common and different elements among D_{Con}, D_{Engy}, and D_{Evol}, as well as (2) to examine differences between interacting vs. non-interacting exon pairs based on secondary structural and IDR contents (Supplementary Section 1.6).

Regarding protein and exon pair overlaps, we find the following. D_{Con} contains the highest number of protein pairs (~50% more protein pairs than D_{Engy} and 140% more protein pairs than D_{Evol}), showing marginal overlaps with the other two datasets. D_{Engy} contains the highest number of exon pairs, followed by D_{Con}, and D_{Evol}, where each pair of datasets show marginal overlaps (Supplementary Figure S3). Recall that in D_{Con} any two exons are defined to be interacting if at least one residue pair from the two exons is within a predefined distance threshold, which is a weaker EEI definition than the one used for D_{Engy} and D_{Evol}

that incorporate the thermodynamics or the evolutionary conservation, respectively, of the EEI formation (Section 2.1). Similar to what we find for the test protein pairs, D_{Con} has the highest numbers of unique protein and exon pairs in the training sets (Supplementary Figure S4).

Regarding the secondary structural content, for each of D_{Con}, D_{Engy}, and D_{Evol}, we find significant (q-value < 0.05) differences between interacting and non-interacting exon pairs for at least one secondary structural label (Supplementary Figure S5). With respect to α-helix, while for D_{Con} and D_{Engy} there are significant differences between interacting and non-interacting exon pairs, for D_{Evol}, such differences only hold in terms of the Kolmogorov-Smirnov test. With respect to β-sheet, we see significant differences between interacting and non-interacting exon pairs for D_{Con} and D_{Evol}, but not for D_{Engy}. With respect to coil-turn, we see significant differences between interacting and non-interacting exon pairs for D_{Engy} and D_{Evol}, but not for D_{Con}. Although all three secondary structural labels have previously been related to protein complex interfaces (Watkins and Arora, 2014; Gavenonis *et al.*, 2014; Bergey *et al.*, 2013), our results show varied patterns of differences across D_{Con}, D_{Engy}, and D_{Evol}, potentially influenced by the different EEI definitions used.

Regarding the IDR content, we find significant (q-value < 0.05) differences for D_{Con} and D_{Engy}, with interacting exon pairs having more IDR content (Supplementary Figure S5, last row). For D_{Evol}, we do not find a statistically significant difference. Because interacting exon pairs are expected to have more of their regions on the surface (rather than the core) of proteins compared to non-interacting exon pairs, and because IDRs are more likely to be found on protein surfaces than protein cores (van der Lee *et al.*, 2014; Wright and Dyson, 2015), our results above are in-line with previous findings.

Regarding the secondary structural content of the five training sets, for each dataset, we find significant (q-value < 0.05) differences between interacting and non-interacting exon pairs for at least one secondary structural label (Supplementary Figure S6). With respect to α-helix, for D_{Engy}, we find significant differences only in terms of the Kolmogorov-Smirnov test while all other tests show significant differences. With respect to β-sheet, we see significant differences between interacting and non-interacting exon pairs for D_{Con} and D_{Engy}, but not for D_{Evol}. With respect to coil-turn, we see significant differences between interacting and non-interacting exon pairs for all datasets. Regarding the IDR content, we find significant (q-value < 0.05) differences between interacting and non-interacting exon pairs, with interacting exon pairs having more IDR content (Supplementary Figure S6, last row).

To summarize, across D_{Con}, D_{Engy}, and D_{Evol}, we see differences not only in terms of the number of data points (i.e., protein or exon pairs) but also in terms of the 3D structural content of interacting vs. non-interacting exon pairs. Because we use each of D_{Con}, D_{Engy}, and D_{Evol} to evaluate the considered PPIIP methods in the subsequent sections in the main paper (Sections 3.2-3.5), the above data differences could also percolate into the performance differences of a PPIIP method across D_{Con}, D_{Engy}, and D_{Evol}.

1.7.2 Statistical evaluation of the PPIIP methods on the RRI prediction task

We statistically quantify how well the methods perform in comparison to one another (Supplementary Section 1.5.2). Given an FDR choice, we compare a method to each of the remaining three methods using each of the four threshold-dependent performance measures, resulting in 12 comparisons. We find that dMaSIF shows significantly better (q-value < 0.05) performance in 5, 8, 10, 12, and 12 comparisons for the FDR choice of 1%, 2%, 3%, 4%, and 5%, respectively. PInet shows significantly better performance in three comparisons for the FDR choice of 1%, while GLINTER shows significantly better performance in only two comparisons for the same FDR choice. ProteinMAE does not show significantly better performance in any of the 12 comparisons for any FDR choice (Supplementary Table S4a).

1.7.3 Statistical evaluation of the PPIIP methods on the EEI prediction task

With respect to each of AU-ROC and AU-PRC, for each of the PPIIP methods, when compared across all 15 combinations of the three datasets and the five test sets, PPDL performs better than PPMax, although not statistically significant (q-value < 0.05) (Supplementary Figure S9). With respect to MCC, precision, recall, and F-score, for a PPIIP method, when compared across all 15 combinations of the three datasets and the five test sets at a given FDR choice, we find the following. For MCC, the performance significance (q-value < 0.05) of PPDL over PPMax exists for ProteinMAE at each of the FDR choices, for PInet at the FDR choices of 1% and 2%, while for dMaSIF and GLINTER there is no significant difference between PPDL and PPMax for any FDR choice (Supplementary Figure S9). For precision, for each of the PPIIP methods, although PPDL on average performs better than PPMax in most cases, the differences in the performances are non-significant (Supplementary Figure S9). For recall and F-score, the performance significance of PPDL over PPMax varies depending on the PPIIP method and FDR choice, as follows. While for dMaSIF and ProteinMAE, PPDL significantly (q-value < 0.05) outperforms PPMax at each of the FDR choices, for PInet, PPDL significantly outperforms PPMax at each of the FDR choices except 4%. For GLINTER, there is no significant difference between PPDL and PPMax (Supplementary Figure S9).

2 Supplementary Figures

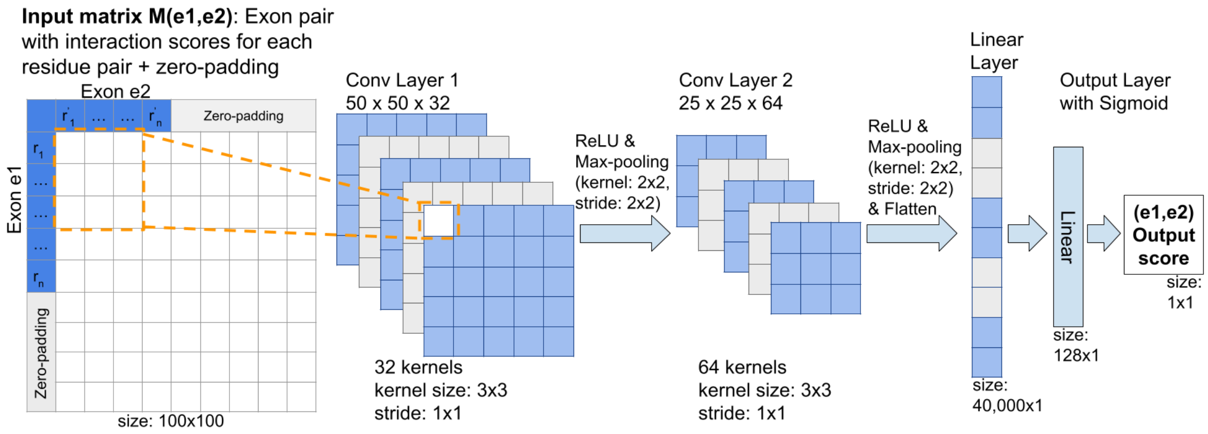

Supplementary Figure S1: Our PPDL architecture. The input is an exon pair with RRI prediction scores for each pair of residues across the exons. The output is a score between 0 and 1. The architecture has two convolutional layers, two max-pooling, two non-linear activations, and two linear layers for final classification. The layers are represented using abbreviations such as 'Conv' for convolutional layers, 'ReLU' for activation with Rectified Linear Unit, and 'Max-pooling' for max-pooling layers. For each layer, if applicable, we write the number of kernels, kernel size, and stride. The final layer utilizes the 'Sigmoid' activation function. To train our PPDL architecture, we minimize the binary cross entropy loss using the Adam optimizer (Kingma and Ba, 2014) and an initial learning rate of 0.0001. We reduce the learning rate every five epochs using a learning rate scheduler. We choose a gamma value of 0.5 for the scheduler so that during training the learning rate is multiplied by 0.5 in every five epochs. This reduction in the learning rate helps to fine-tune the performance of the model by reducing the step size, allowing for convergence to a better solution. To reduce overfitting, we set the maximum number of epochs to 20 including early stopping.

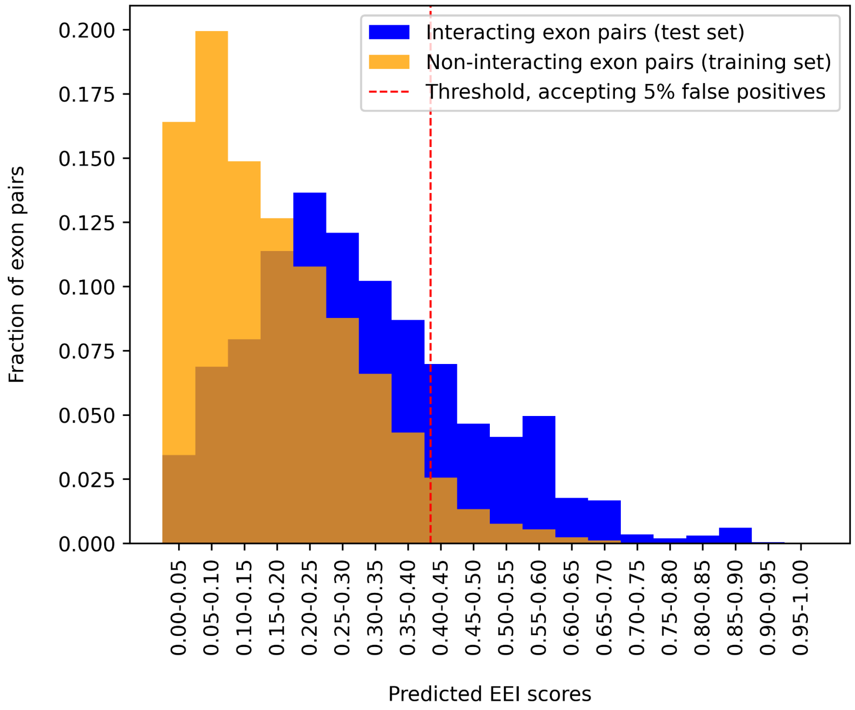

Supplementary Figure S2: Normalized distribution of the number of exon pairs with prediction scores for non-interacting exon pairs (orange), and interacting exon pairs (blue). A bin shows the fraction of exon pairs (y-axis) with prediction scores within the corresponding score range (x-axis). We take the predictions of all non-interacting exon pairs from the training set as our background (or null) distribution. Then, we define a decision threshold (red line) as the score that accepts 5% of non-interacting exon pairs (i.e., false positives) from this background, which corresponds to an FDR of 5%. This is an example plot for a test dataset of the D_{Con} dataset corresponding to the dMaSIF+PPDL approach for EEI prediction.

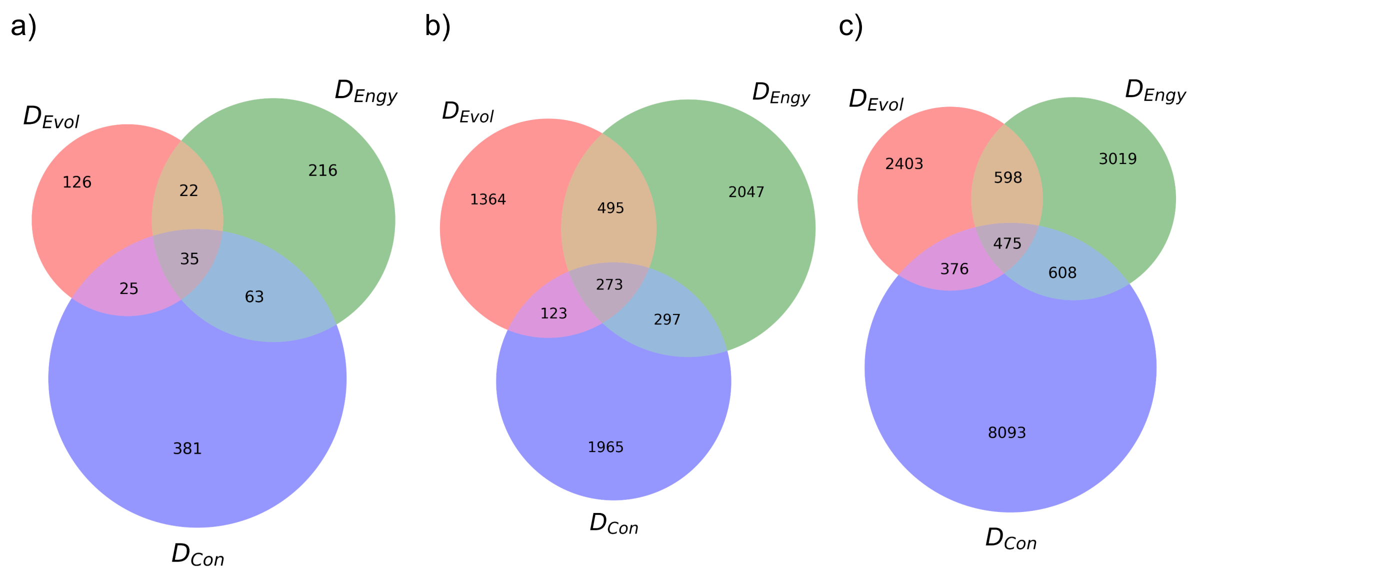

Supplementary Figure S3: Venn diagrams depicting overlaps between test sets of D_{Con}, D_{Engy}, and D_{Evol} in terms of (a) protein pairs, (b) interacting exon pairs, and (c) non-interacting exon pairs.

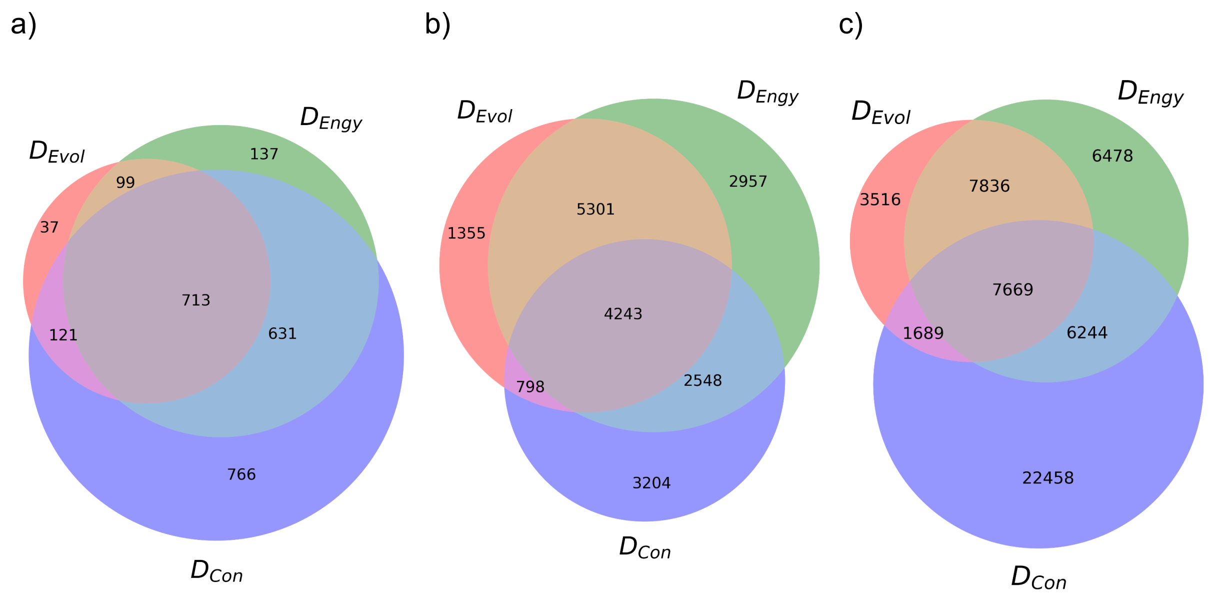

Supplementary Figure S4: Venn diagram depicting overlaps between training sets of D_{Con}, D_{Engy}, and D_{Evol} in terms of (a) protein pairs, (b) interacting exon pairs, and (c) non-interacting exon pairs.

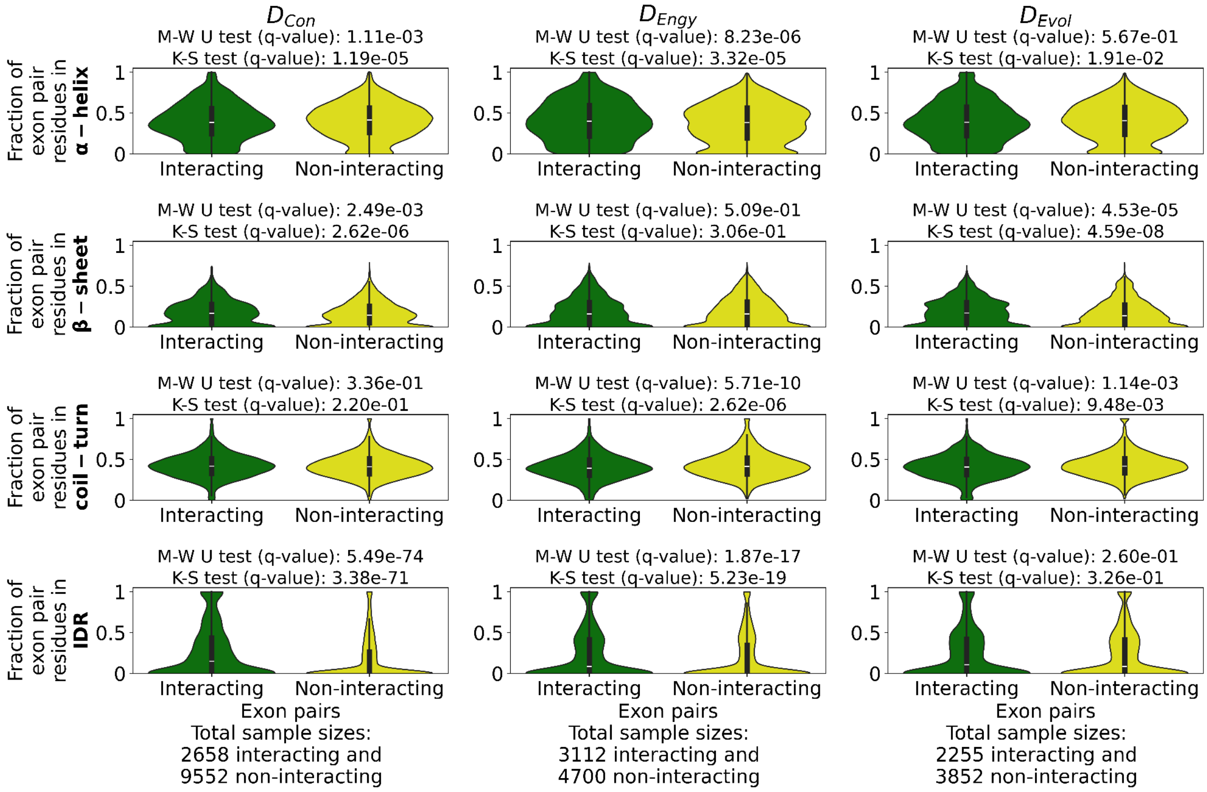

Supplementary Figure S5: Violin plots depicting the distribution of secondary structural labels, i.e., α-helix, β-sheet, or coil-turn, and IDR (rows) in interacting vs. non-interacting exon pairs of test sets across D_{Con}, D_{Engy}, and D_{Evol} (columns).

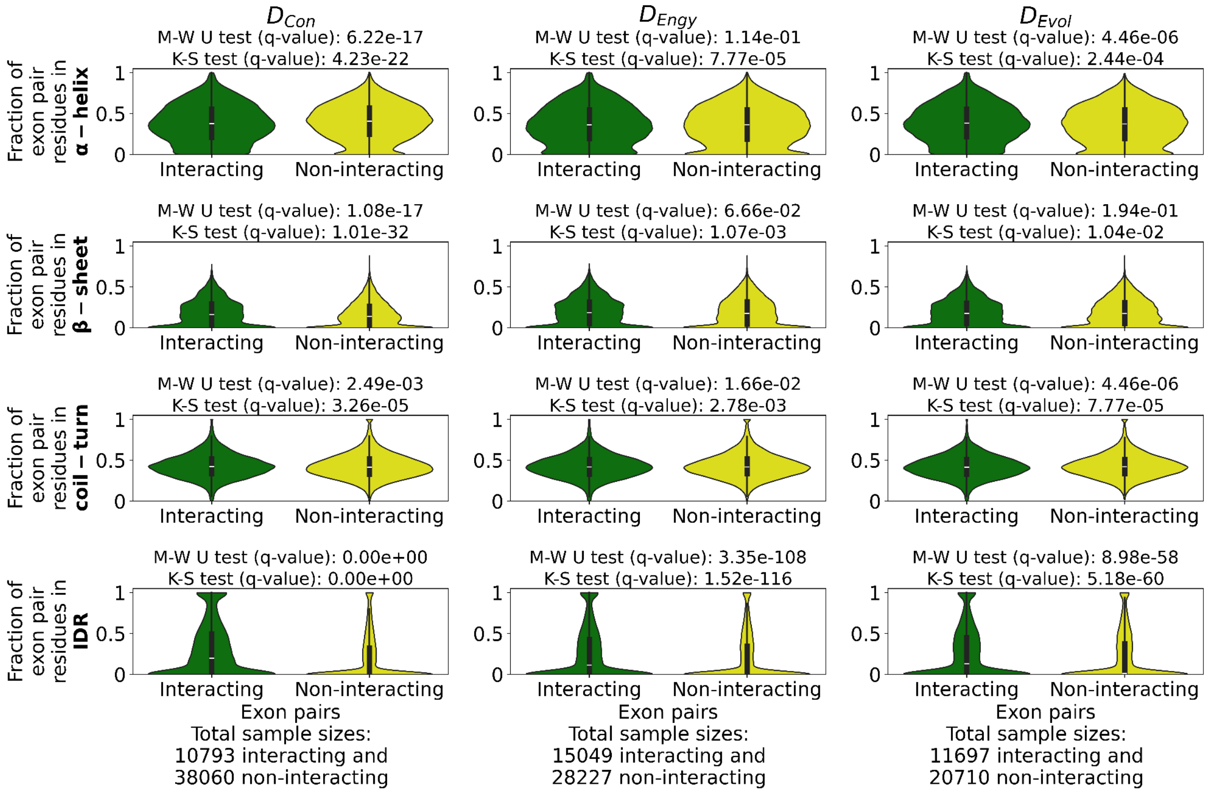

Supplementary Figure S6: Violin plots depicting the distribution of secondary structural labels, i.e., α-helix, β-sheet, or coil-turn, and IDRs (rows) in interacting vs. non-interacting exon pairs of training sets across the three datasets D_{Con}, D_{Engy}, and D_{Evol} (columns).

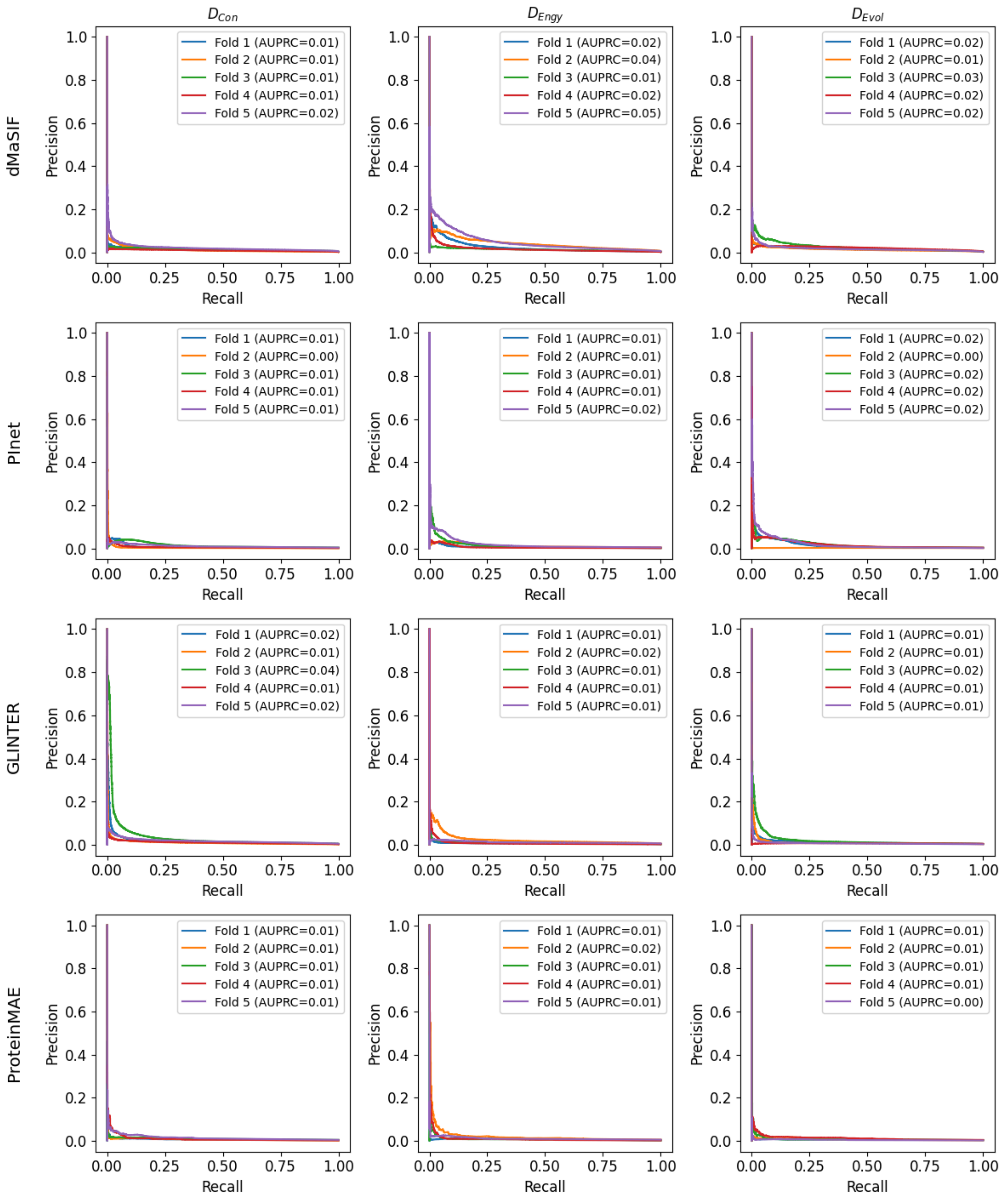

Supplementary Figure S7: Precision-recall (PR) curves of the four PPIIP methods (rows: dMaSIF, PInet, GLINTER, ProteinMAE) across the three datasets (columns: D_{Con}, D_{Engy}, and D_{Evol}) for the RRI predictions. Each subfigure corresponds to one method-dataset combination and shows five PR curves, one for each of the five test sets. We show the values for the area under the PR curve in the corresponding legend.

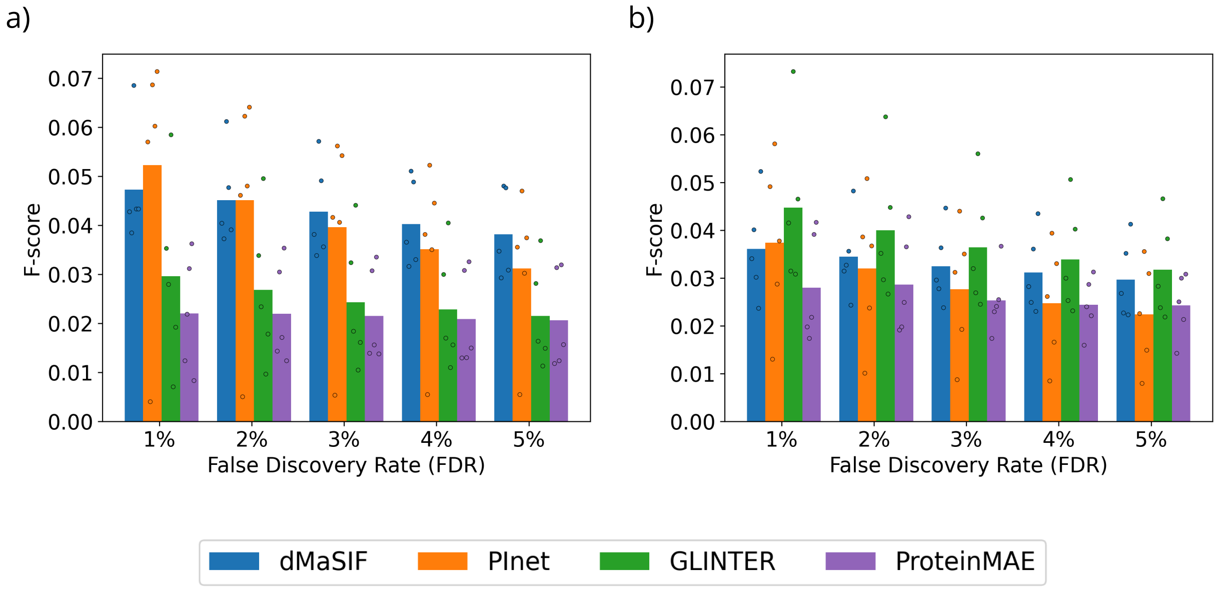

Supplementary Figure S8: F-scores of the PPIIP methods on the RRI prediction task for multiple FDRs for the a) D_{Evol} dataset and b) D_{Con} dataset. The height of a given bar represents the mean F-score across the five independent test sets, while the individual data points indicate the F-score for each test set.

Performance measures	FDR	dMaSIF	PInet	GLINTER	ProteinMAE
MCC	1%	0.20686	0.01460	0.54642	0.00674
MCC	2%	0.14769	0.00674	0.77173	0.00531
MCC	3%	0.33312	0.10688	0.87430	0.00531
MCC	4%	0.40661	0.33312	0.98934	0.00590
MCC	5%	0.42257	0.25059	0.71826	0.00590
F-score	1%	0.00674	0.00815		0.00674
F-score	2%	0.00631	0.00531	0.39117	0.00674
F-score	3%	0.00674	0.01460	0.42257	0.00531
F-score	4%	0.00631	0.02478	0.63459	0.00590
F-score	5%	0.00674	0.01718	0.40661	0.00531
Precision	1%	0.77173	0.13199	0.52074	0.98934
Precision	2%	0.98934	0.22799	0.86044	0.86044
Precision	3%	0.74508	0.86044	1.00000	0.44649
Precision	4%	0.88728	0.87430	0.98934	0.54642
Precision	5%	0.79802	0.87430	0.79802	0.92866
Recall	1%	0.00674	0.00974	0.40661	0.00674
Recall	2%	0.00631	0.00631	0.42257	0.00974
Recall	3%	0.00631	0.01718	0.44649	0.00590
Recall	4%	0.00631	0.05160	0.56376	0.00674
Recall	5%	0.00815	0.02478	0.56376	0.00631
AUROC		0.59883	0.09446	0.11747	0.74508
AUPRC		0.56376	0.11747	0.36151	0.16461

PPIIP methods

Supplementary Figure S9: Statistical comparison of PPMax and PPDL. For each PPIIP method, we compare performance for PPMax and PPDL using the Wilcoxon signed-rank test across all combinations of dataset, performance measure, and FDR choice (where applicable). The resulting p-values are corrected for multiple testing using the Benjamini-Hochberg procedure to obtain q-values. We highlight cells in orange where PPDL significantly (q-value < 0.05) outperforms PPMax. There is no significant difference in favor of PPMax. One cell is left empty because no true positives are observed, making the F-score undefined.

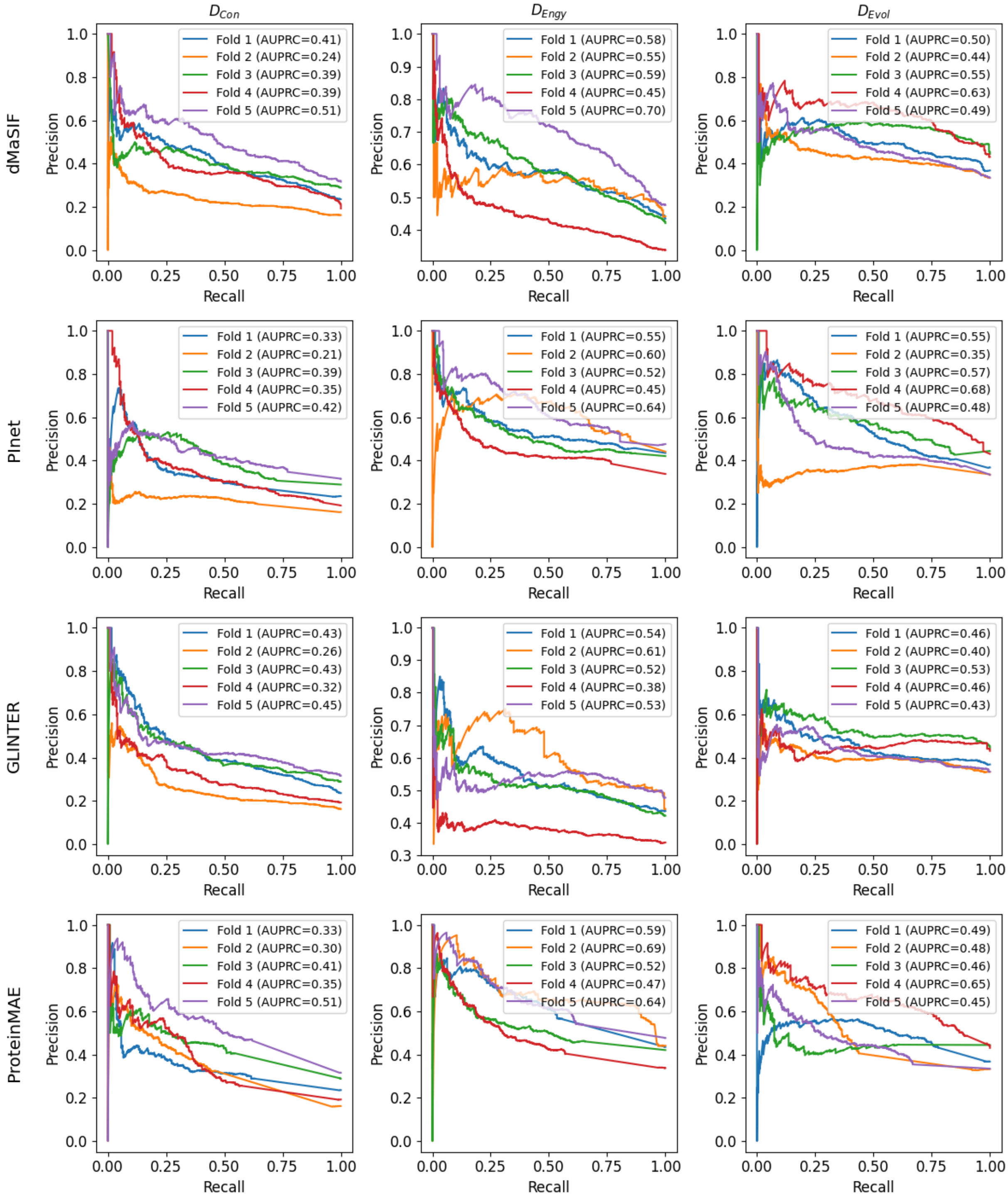

Supplementary Figure S10: Precision-recall (PR) curves of the four PPIIP methods (rows: dMaSIF, PInet, GLINTER, ProteinMAE) across the three datasets (columns: D_{Con}, D_{Engy}, and DEvol) for the EEI predictions using PPDL. Each subfigure corresponds to one method-dataset combination and shows five PR curves, one for each of the five independent test folds. We show the values for the area under the PR curve in the corresponding legend.

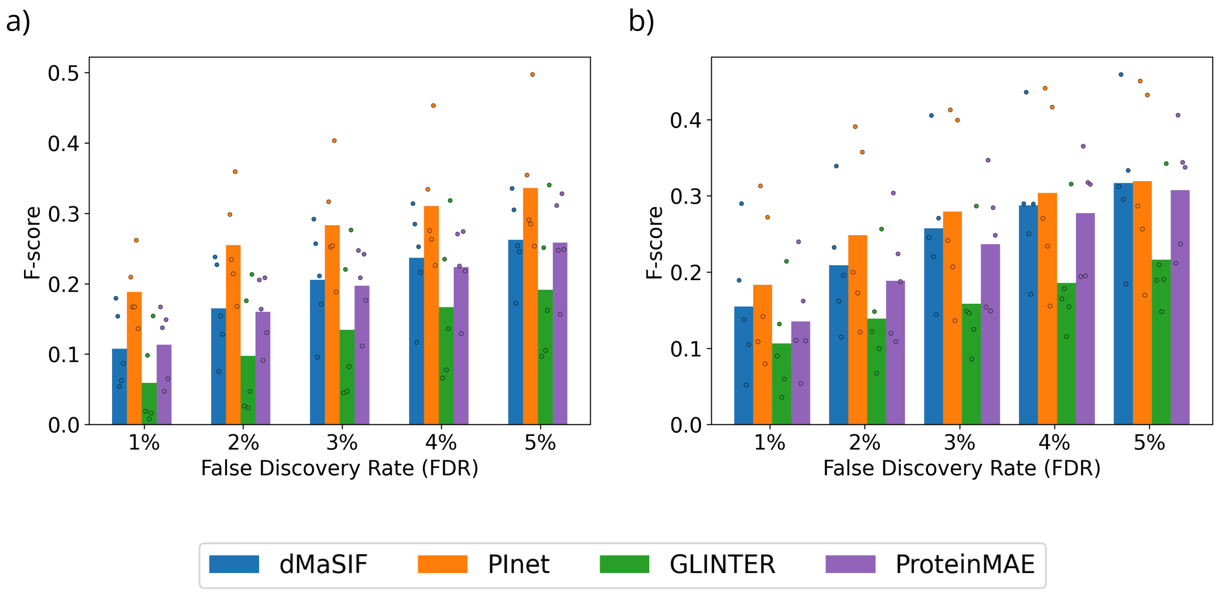

Supplementary Figure S11: F-scores of the PPIIP methods on the EEI prediction task for multiple FDRs for the a) D_{Evol} dataset and b) D_{Engy} dataset. The height of a given bar represents the mean F-score across the five independent test sets, while the individual data points indicate the F-score for each test set.

Performance metrics	FDR	dMaSIF	PInet	GLINTER	ProteinMAE
MCC	1%	0.00410	0.00019	0.00630	0.00010
MCC	2%	0.00203	0.00010	0.00261	0.00010
MCC	3%	0.00157	0.00010	0.00157	0.00010
MCC	4%	0.00027	0.00010	0.00027	0.00010
MCC	5%	0.00019	0.00010	0.00010	0.00019
F-score	1%	0.00060	0.00010	0.00508	0.00010
F-score	2%	0.00010	0.00010	0.00044	0.00010
F-score	3%	0.00010	0.00010	0.00010	0.00010
F-score	4%	0.00010	0.00010	0.00010	0.00010
F-score	5%	0.00010	0.00010	0.00010	0.00010
Precision	1%	0.00010	0.00010	0.00010	0.00010
Precision	2%	0.00010	0.00010	0.00010	0.00010
Precision	3%	0.00010	0.00010	0.00010	0.00010
Precision	4%	0.00010	0.00010	0.00010	0.00010
Precision	5%	0.00010	0.00010	0.00010	0.00010
Recall	1%	0.02092	0.97797	0.00261	0.02885
Recall	2%	0.00060	0.71109	0.00410	0.05337
Recall	3%	0.00019	0.78841	0.00508	0.02885
Recall	4%	0.00010	0.90062	0.00330	0.14891
Recall	5%	0.00010	0.86662	0.00330	0.20384
AUROC		0.00010	0.67715	0.00010	0.67715
AUPRC		0.00010	0.00010	0.00010	0.00010

PPIIP methods

Supplementary Figure S12: Statistical comparison of RRI and EEI using PPDL. For each PPIIP method, we compare the performances for RRI and EEI using PPDL using the Wilcoxon signed-rank test across all combinations of dataset, performance measure, and FDR choice (where applicable). The resulting p-values are corrected for multiple testing using the Benjamini-Hochberg procedure to obtain q-values. We highlight cells in orange where EEI using PPDL significantly (q-value < 0.05) outperforms RRI and in blue where RRI significantly (q-value < 0.05) outperforms EEI using PPDL.

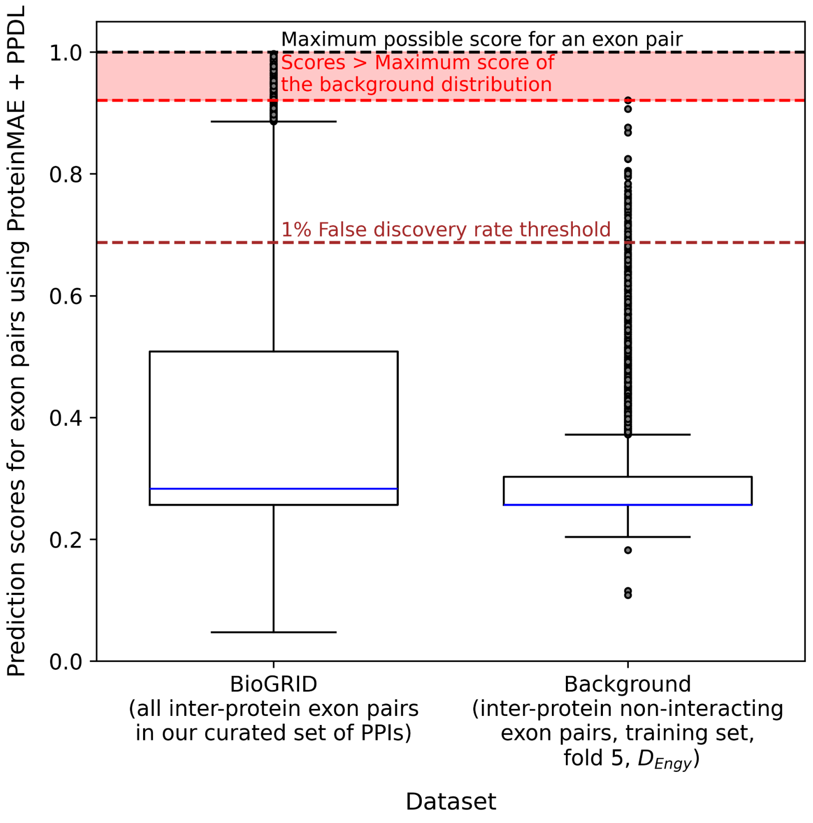

Supplementary Figure S13: Prediction score distributions for all exon pairs in our curated set of PPIs (Supplementary Section 1.1.6) and non-interacting exon pairs from the background distribution, using ProteinMAE with PPDL. Within the boxplot, we compare the prediction scores (y-axis) assigned by ProteinMAE with PPDL to exon pairs of the curated PPIs from BioGRID and to non-interacting exon pairs from the background distribution of the selected pre-trained model (ProteinMAE with PPDL trained on D_{Engy}, fold 5) (x-axis) (Section 2.4, Section 3.4). For each of the two datasets, the median is indicated by a blue line. The horizontal dashed brown line marks the 1% FDR choice derived from the background distribution, the horizontal dashed red line corresponds to the maximum score observed in the background, and the horizontal dashed black line indicates the maximum possible score of 1.0. We highlight in light red the area that contains exon pairs from our curated set BioGRID PPIs with prediction scores higher than the maximum value observed in the background distribution.

3 Supplementary Tables

Supplementary Table S1: List of potential PPIIP methods (in alphabetical order of the method’s name) showing in four columns the (1) title of the publication, (2) name, (3) code availability, and (4) further information. In bold and red we highlight the methods which we use in our study.

**Title of the publication**	**Name of method**	**Code for our use case?**	**Further information**
Prediction of inter-chain distance maps of protein complexes with 2D attention-based deep neural networks (Guo *et al.*, 2022)	CDPred	Yes	CCMpred, and PSSM, two time-consuming pre-processing steps are included.
DeepHomo2.0: improved protein-protein contact prediction of homodimers by transformer-enhanced deep learning (Lin *et al.*, 2022)	DeepHomo2.0	Yes to download	The preprocessing steps (same for DeepInter) are very time-consuming, see Supplementary Table S8.
Protein-protein contact prediction by geometric triangle-aware protein language models (Lin, Tao, *et al.*, 2023)	DeepInter	Yes to download	Very time-consuming preprocessing steps, prediction time for 10 protein pairs: 2.98 hours, see Supplementary Table S8.
Deep transfer learning for inter-chain contact predictions of transmembrane protein complexes (Lin, Yan, *et al.*, 2023)	DeepTMP	No	The preprocessing steps (same for DeepInter) are very time-consuming, see Supplementary Table S8.
**Fast end-to-end learning on protein surfaces** (Sverrisson *et al.*, 2021)	**dMaSIF**	**Yes**	
**Deep graph learning of inter-protein contacts** (Xie and Xu, 2022)	**GLINTER**	**Yes**	
A variational expectation-maximization framework for balanced multi-scale learning of protein and drug interactions (Rao *et al.*, 2024)	MUSE	No	Code is only available for PPI, but not RRI prediction
**Protein interaction interface region prediction by geometric deep learning** (Dai and Bailey-Kellogg, 2021)	**PInet**	**Yes**	
Protein language model-embedded geometric graphs power inter-protein contact prediction (Si and Yan, 2024)	PLMGraph-Inter	Yes	See Supplementary Table S8, codeocean capsule, inference for one protein pair runs longer than 2 hours.
**ProteinMAE: masked autoencoder for protein surface self-supervised learning** (Yuan *et al.*, 2023)	**ProteinMAE**	**Yes**	

Supplementary Table S2: Performance of dMaSIF, PInet, GLINTER, and ProteinMAE on the three datasets (i.e. D_{Con}, D_{Engy}, and D_{Evol}) for RRI predictions with our default interacting/non-interacting threshold of 6 Å. We show the results for the FDRs ranging from 1% to 5% in increments of 1% (Section 2.4), along with AU-ROC and AU-PRC scores. We highlight the best performance across datasets and PPIIP methods per performance measure in red. We present the same results for the threshold 4 Å in Supplementary Table S2-4Å and for the threshold 8 Å Supplementary Table S2-8Å.

Supplementary Table S3: Summary of our datasets. We show the numbers of interacting and non-interacting residue/exon pairs per dataset. In addition, we show the number of exon pairs in each dataset when we only consider exons of length (number of residues) 100 or less.

Supplementary Table S4: Pairwise statistical comparison of PPIIP methods across performance measures and thresholds for (a) RRI prediction and (b) EEI prediction using PPDL. Within (a) and (b), results are shown in the following order: MCC, F-score, precision, and recall at FDR = 5%, followed by AU-ROC and AU-PRC. Subsequently, we show results for MCC, F-score, precision, and recall at FDR = 1%, 2%, 3%, and 4%. In each subtable, every cell (i, j) indicates the statistical significance (in terms of q-value) of approach i (rows) being superior to approach j (columns). We highlight in green all q-value < 0.05.

Supplementary Table S5: Performances of dMaSIF, PInet, GLINTER, and ProteinMAE on the three datasets (i.e. D_{Con}, D_{Engy}, and D_{Evol}) on the EEI prediction task using PPMax. We show the results for five FDR choices ranging from 1% to 5% in increments of 1% (Section 2.4), along with AU-ROC and AU-PRC scores. We highlight the best performance across datasets and PPIIP methods per performance measure in red.

Supplementary Table S6: Performances of dMaSIF, PInet, GLINTER, and ProteinMAE on the three datasets (i.e. D_{Con}, D_{Engy}, and D_{Evol}) on the EEI prediction task using PPDL. We show the results for five FDR choices ranging from 1% to 5% in increments of 1 (Section 2.4), along with AU-ROC and AU-PRC scores. We highlight the best performance across datasets and methods per performance measure in red.

Supplementary Table S7:List of the prediction scores from ProteinMAE with PPDL for all exon pairs mapped to their UniProt protein IDs in our BioGRID-derived dataset. Exon pairs with scores higher than the maximum background score are highlighted in green. Those with scores between the 1% FDR choice and the background maximum are highlighted in yellow.

Supplementary Table S8: List of proteins used for the computational runtime evaluations of the four PPIIP methods and the runtime (Section 3.8). We also include the runtime of the preprocessing steps required by DeepInter, and report the runtime of DeepGraph-Inter.

References:

Altschul,S.F. *et al.* (1990) Basic local alignment search tool. *J. Mol. Biol.*, **215**, 403–410.

Aspromonte,M.C. *et al.* (2024) DisProt in 2024: improving function annotation of intrinsically disordered proteins. *Nucleic Acids Res.*, **52**, D434–D441.

Baker,N.A. *et al.* (2001) Electrostatics of nanosystems: application to microtubules and the ribosome. *Proc. Natl. Acad. Sci. U. S. A.*, **98**, 10037–10041.

Bergey,C.M. *et al.* (2013) HippDB: a database of readily targeted helical protein-protein interactions. *Bioinformatics*, **29**, 2806–2807.

Berman,H.M. *et al.* (2000) The Protein Data Bank. *Nucleic Acids Res.*, **28**, 235–242.

Bernett,J. *et al.* (2024) Cracking the black box of deep sequence-based protein–protein interaction prediction. **25**, bbae076.

Bliven,S. *et al.* (2018) Automated evaluation of quaternary structures from protein crystals. *PLoS Comput. Biol.*, **14**, e1006104.

Dai,B. and Bailey-Kellogg,C. (2021) Protein interaction interface region prediction by geometric deep learning. *Bioinformatics*, **37**, 2580–2588.

Dana,J.M. *et al.* (2019) SIFTS: updated Structure Integration with Function, Taxonomy and Sequences resource allows 40-fold increase in coverage of structure-based annotations for proteins. *Nucleic Acids Res.*, **47**, D482–D489.

Dawson-Haggerty,M. trimesh, 2019. *URL https://github. com/mikedh/trimesh*.

DeLano,W.L. and Others (2002) Pymol: An open-source molecular graphics tool. *CCP4 Newsl. Protein Crystallogr*, **40**, 82–92.

Dolinsky,T.J. *et al.* (2007) PDB2PQR: expanding and upgrading automated preparation of biomolecular structures for molecular simulations. *Nucleic Acids Res.*, **35**, W522–5.

Durinck,S. *et al.* (2005) BioMart and Bioconductor: a powerful link between biological databases and microarray data analysis. *Bioinformatics*, **21**, 3439–3440.

Fukuchi,S. *et al.* (2012) IDEAL: Intrinsically Disordered proteins with Extensive Annotations and Literature. *Nucleic Acids Res.*, **40**, D507–11.

Gavenonis,J. *et al.* (2014) Comprehensive analysis of loops at protein-protein interfaces for macrocycle design. *Nat. Chem. Biol.*, **10**, 716–722.

Guo,Z. *et al.* (2022) Prediction of inter-chain distance maps of protein complexes with 2D attention-based deep neural networks. *Nature Communications*, **13**.

H. Pagès, P. Aboyoun, R. Gentleman, and S. DebRoy (2017) Biostrings Bioconductor.

Kabsch,W. and Sander,C. (1983) Dictionary of protein secondary structure: pattern recognition of hydrogen-bonded and geometrical features. *Biopolymers*, **22**, 2577–2637.

Kingma,D.P. and Ba,J. (2014) Adam: A Method for Stochastic Optimization. *arXiv [cs.LG]*.

Krissinel,E. and Henrick,K. (2007) Inference of macromolecular assemblies from crystalline state. *J. Mol. Biol.*, **372**, 774–797.

van der Lee,R. *et al.* (2014) Classification of intrinsically disordered regions and proteins. *Chem. Rev.*, **114**, 6589–6631.

Lin,P. *et al.* (2022) DeepHomo2.0: improved protein-protein contact prediction of homodimers by transformer-enhanced deep learning. *Briefings in bioinformatics*.

Lin,P., Yan,Y., *et al.* (2023) Deep transfer learning for inter-chain contact predictions of transmembrane protein complexes. *Nat. Commun.*, **14**, 1–16.

Lin,P., Tao,H., *et al.* (2023) Protein–protein contact prediction by geometric triangle-aware protein language models. *Nature Machine Intelligence*, **5**, 1275–1284.

Majila,K. and Viswanath,S. (2024) StrIDR: a database of intrinsically disordered regions of proteins with experimentally resolved structures. *bioRxiv*, 2024.08.22.609111.
Martin,F.J. *et al.* (2023) Ensembl 2023. *Nucleic Acids Res.*, **51**, D933–D941.
Massey,F.J. (1951) The Kolmogorov-Smirnov Test for Goodness of Fit. *J. Am. Stat. Assoc.*, **46**, 68–78.
McKnight,P.E. and Najab,J. (2010) Mann-Whitney U Test. *The Corsini Encyclopedia of Psychology*.
Mirdita,M. *et al.* (2016) Uniclust databases of clustered and deeply annotated protein sequences and alignments. *Nucleic Acids Res.*, **45**, D170–D176.
Oughtred,R. *et al.* (2021) The BioGRID database: A comprehensive biomedical resource of curated protein, genetic, and chemical interactions. *Protein Sci.*, **30**, 187–200.
Paszke,A. *et al.* (2019) PyTorch: An Imperative Style, High-Performance Deep Learning Library. In, Wallach,H. *et al.* (eds), *Advances in Neural Information Processing Systems*. Curran Associates, Inc.
Piovesan,D. *et al.* (2023) MobiDB: 10 years of intrinsically disordered proteins. *Nucleic Acids Res.*, **51**, D438–D444.
Rao,J. *et al.* (2024) A variational expectation-maximization framework for balanced multi-scale learning of protein and drug interactions. *Nat. Commun.*, **15**, 1–12.
Rao,R.M. *et al.* (18--24 Jul 2021) MSA Transformer. In, Meila,M. and Zhang,T. (eds), *Proceedings of the 38th International Conference on Machine Learning*., pp. 8844–8856.
Rost,B. (1999) Twilight zone of protein sequence alignments. *Protein Eng.*, **12**, 85–94.
Sanner,M.F. *et al.* (1996) Reduced surface: an efficient way to compute molecular surfaces. *Biopolymers*, **38**, 305–320.
Si,Y. and Yan,C. (2024) Protein language model-embedded geometric graphs power inter-protein contact prediction. *Elife*, **12**, RP92184.
Steinegger,M. and Söding,J. (2017) MMseqs2 enables sensitive protein sequence searching for the analysis of massive data sets. *Nat. Biotechnol.*, **35**, 1026–1028.
Sverrisson,F. *et al.* (2021) Fast end-to-end learning on protein surfaces. In, *2021 IEEE/CVF Conference on Computer Vision and Pattern Recognition (CVPR)*. IEEE.
UniProt Consortium (2019) UniProt: a worldwide hub of protein knowledge. *Nucleic Acids Res.*, **47**, D506–D515.
Varadi,M. *et al.* (2022) AlphaFold Protein Structure Database: massively expanding the structural coverage of protein-sequence space with high-accuracy models. *Nucleic Acids Res.*, **50**, D439–D444.
Velankar,S. *et al.* (2013) SIFTS: Structure Integration with Function, Taxonomy and Sequences resource. *Nucleic Acids Res.*, **41**, D483–9.
Watkins,A.M. and Arora,P.S. (2014) Anatomy of β-strands at protein-protein interfaces. *ACS Chem. Biol.*, **9**, 1747–1754.
Word,J.M. *et al.* (1999) Asparagine and glutamine: using hydrogen atom contacts in the choice of side-chain amide orientation. *J. Mol. Biol.*, **285**, 1735–1747.
Wright,P.E. and Dyson,H.J. (2015) Intrinsically disordered proteins in cellular signalling and regulation. *Nat. Rev. Mol. Cell Biol.*, **16**, 18–29.
Xie,Z. and Xu,J. (2022) Deep graph learning of inter-protein contacts. *Bioinformatics*, **38**, 947–953.
Yuan,M. *et al.* (2023) ProteinMAE: masked autoencoder for protein surface self-supervised learning. *Bioinformatics*, **39**, btad724.
